# Topolow: a mapping algorithm for antigenic cross-reactivity and binding affinity assays

**DOI:** 10.1093/bioinformatics/btaf372

**Published:** 2025-06-25

**Authors:** Omid Arhami, Pejman Rohani

**Affiliations:** Institute of Bioinformatics, University of Georgia, Athens, GA 30602-7229, United States; Center for Influenza Disease & Emergence Research (CIDER), University of Georgia, Athens, GA 30602, United States; Institute of Bioinformatics, University of Georgia, Athens, GA 30602-7229, United States; Center for Influenza Disease & Emergence Research (CIDER), University of Georgia, Athens, GA 30602, United States; Odum School of Ecology, University of Georgia, Athens, GA 30602, United States

## Abstract

**Motivation:**

Understanding antigenic evolution through cross-reactivity assays is crucial for tracking rapidly evolving pathogens requiring regular vaccine updates. However, existing cartography methods, commonly based on multidimensional scaling (MDS), face significant challenges with sparse and complex data, producing incomplete and inconsistent maps. There is an urgent need for robust computational methods that can accurately map antigenic relationships from incomplete experimental data while maintaining biological relevance, especially given that more than 95% of possible measurements could be missing in large-scale studies.

**Results:**

We present Topolow, an algorithm that transforms cross-reactivity and binding affinity measurements into accurate positions in a phenotype space. Using a physics-inspired model, Topolow achieved comparable prediction accuracy to MDS for H3N2 influenza and 56% and 41% improved accuracy for dengue and HIV, while maintaining complete positioning of all antigens. The method effectively reduces experimental noise and bias, determines optimal dimensionality through likelihood-based estimation, avoiding distortions due to insufficient dimensions, and demonstrates orders of magnitude better stability across multiple runs. We also introduce antigenic velocity vectors, which measure the rate of antigenic advancement of each isolate per unit of time against its temporal and evolutionary related background, revealing the underlying antigenic relationships and cluster transitions.

**Availability and implementation:**

Topolow is implemented in R and freely available at https://doi.org/10.5281/zenodo.15620983 and https://github.com/omid-arhami/topolow.

## 1 Introduction

Understanding and tracking the antigenic evolution of viruses is crucial for public health, particularly for rapidly evolving pathogens like influenza that require regular vaccine updates (Smith *et al.* 2004, Koel *et al.* 2013, Neher *et al.* 2016). The ability of viruses to escape immune recognition through antigenic change enables them to reinfect previously exposed individuals (Harvey *et al.* 2016). This process, known as antigenic drift, is quantified through cross-reactivity assays that measure how well antibodies generated against one virus isolate recognize and neutralize other strains (Hirst 1943, Hensley *et al.* 2009). Assay outcomes are expressed as either titers (highest dilution producing measurable effect) or concentrations (e.g. $IC_{50}$ for 50% inhibition).

Given the resource-intensive nature and substantial costs of these assays, only a small fraction of possible pairwise antigenic relationships are measured directly. Assay measurements also exhibit significant experimental variability (Zacour *et al.* 2016). This set of non-complete and noisy pairwise immunological measurements forms a complex network of relationships that can be difficult to interpret directly.

The development of antigenic cartography, primarily through multidimensional scaling (MDS), has been instrumental in visualizing and analysing antigenic relationships among viruses (Smith *et al.* 2004). MDS-based methods project immunological measurements into a continuous low-dimensional space where antigenic distances between viruses correspond to their immunological differences. A compelling demonstration of the MDS approach to antigenic cartography was provided by Smith *et al.* (2004), who used gradient descent to minimize the sum of squared errors between predicted and measured distances. It is implemented in the R package RACMACS (Wilks 2024). The method does, however, face challenges with sparse data (Bravo 2002)—a common issue as dataset size increases (Cai *et al.* 2010).

Data sparsity arises since assays such as hemagglutination inhibition (HI) are typically constrained to a limited number of contemporary antigens. When separate HI tables are merged into a super-matrix, the resulting table is generally highly incomplete, with up to 95% missing values in datasets spanning multiple decades (Cai *et al.* 2010). The abundance of missing values in a dataset forms one of the most significant barriers to creating accurate antigenic maps (Bravo 2002, Cai *et al.* 2010).

Thinking of cartography from a different perspective helps understand the problem of missing measurements. Creating a map is equivalent to determining the coordinates of the points in an *r*-dimensional space, where *r* can be any integer greater than 1. If we have *c* points and their similarities to, or distances from, *r* references are fully measured, the coordinates of points can be exactly determined in an *r*-dimensional space. However, if only a part of similarities/distances between points and references are available, each point can assume an infinite number of positions in the *r*-dimensional space, and there will not be a unique solution. In this case, the common approach is to use MDS to find the positions in a lower-dimension space (Lapedes and Farber 2001). However, missing data creates a challenging tradeoff for MDS between accuracy and completeness in dimensionality selection: choosing more dimensions for the target space increases accuracy but hinders finding positions for all points when the number of measurements is smaller than the number of dimensions. Conversely, choosing lower dimensionality causes loss of information and compromises the accuracy of the estimated positions. As the proportion of missing data increases, the dimensionality selection becomes more challenging due to insufficient constraints from the observed distances. Furthermore, as a gradient-based algorithm, RACMACS implementation of MDS is adversely affected by missing data, which impact the accuracy of the magnitude and directions of gradient vectors.

Additionally, as we demonstrate in this article, antigenic maps created by MDS for the same data vary substantially between runs. This convergence instability, combined with relatively high mapping errors, affects our ability to visualize accurately and understand antigenic evolution with confidence, ultimately impacting critical public health decisions in vaccine development and viral surveillance efforts.

Several variants have been developed to improve performance relative to MDS in the context of antigenic data, such as use of non-metric MDS (Lapedes and Farber 2001), temporal matrix completion (Cai *et al.* 2010), Bayesian MDS (Bedford *et al.* 2014), biological matrix completion (Huang *et al.* 2017), and integrating protein structure (Harvey *et al.* 2023).

Here, we adopt a novel, physics-inspired optimization framework that transforms cross-reactivity titers and binding affinity values into spatial representations in the optimal dimensionality. Our method is called Topological Optimization for Low-Dimensional Mapping or **Topolow**. The algorithm estimates the antigenic map through sequential optimization of pairwise distances, reducing the multidimensional problem to a series of 1D calculations. This gradient-free approach eliminates the need for complex gradient computations required by RACMACS, enabling robust performance even with substantially incomplete assay data. As demonstrated in our results, Topolow achieves superior accuracy compared to MDS when handling missing data.

Antigenic evolution is rarely uniform; some lineages accelerate, whereas others stall or branch (Bush *et al.* 1999, Bedford *et al.* 2014, Neher *et al.* 2016). Therefore, we introduce **antigenic velocity**, a vectorial description of rate and direction of each antigen’s drift. This offers more insight than scalar summaries such as “antigenic advance per year” (e.g. in Neher *et al.* (2016)).

## 2 Materials and methods

### 2.1 Data description and preparation

Pairwise similarity measurements, such as HI titers or neutralization $IC_{50}$ values, are typically represented in matrix form. The matrix contains three types of entries: (i) numeric values, (ii) threshold values that may represent either low (≤h) or high reactors (>h), and (iii) missing values.

A titer $T_{ij}$ in HI assay measures the similarity between test antigen *i* and reference antigen *j*; i.e. transformed into a dissimilarity measure denoted by $D_{ij}$:

(1) $$D_{ij}={\;\text{log}}_{2}(T_{max_{j}})-{\;\text{log}}_{2}(T_{ij}),$$

where $T_{max_{j}}$ represents the maximum titer value observed for the reference antigen (Smith *et al.* 2004). Since experimental conditions and antiserum potency can vary between assay panels, we search the entire dataset for the maximum titer of each reference antigen rather than using either the homologous titer (Neher *et al.* 2016) or the maximum titer within a single panel or clade. This approach helps normalize measurements across different experimental batches.

Threshold values (h) are incorporated in the algorithm as equality constraints. Missing values can be predicted by the model once antigenic coordinates are determined.

### 2.2 Proposed mathematical model

The algorithm models antigenic relationships as a physical system where test and reference antigens are represented as similar particles in an *N*-dimensional space, resembling force-directed graph layout approaches (Kobourov 2012). Each pair of particles for which we have a measurement is connected by a spring with a free length equal to their measured dissimilarity, $D_{ij}$. Following Hooke’s law, the magnitude of the force exerted by the spring is proportional to the displacement from its free length: $F_{s,ij,t}=k(r_{ij}-D_{ij})$, where *k* is the spring constant and $r_{ij}$ is the current distance between particles *i* and *j*.

Pairs of particles lacking a direct measurement apply a repulsive forces to each other that follows the inverse square law: $F_{r,ij,t}=\frac{c}{r_{ij}^{2}}$, where *c* is a repulsion constant that is fitted from data. This design choice is both biologically sensible and computationally efficient. Previous studies have shown that antigenic distances between temporally distant strains tend to be large (Smith *et al.* 2004, Cai *et al.* 2010), yet such pairs are rarely measured in laboratory assays due to logistical constraints. By applying repulsion specifically between pairs with no measurement, our model naturally facilitates separation while avoiding unnecessary force calculations.

For each particle *i*, the total force $F_{i}$ is the sum of spring forces from its measured neighbors ($N_{i}$) and repulsive forces from non-connected particles:

(2) $$F_{i}=-\sum_{j\in N_{i}}k(r_{ij}-D_{ij}){\hat{r}}_{ij}+\sum_{j\notin N_{i}}\left(\frac{c}{r_{ij}^{2}}\right){\hat{r}}_{ij},$$

where ${\hat{r}}_{ij}$ is the unit vector from *i* to *j*.

We assign weights to particles in their motion under the forces, analogous to vertex-weighting schemes used in force-directed graph layouts (Fruchterman and Reingold 1991). Each antigen receives an effective mass $m_{i}$ proportional to its number of measurements, in contrast with traditional MDS which weights all coordinates equally. This weighting scheme provides a natural regularization, preventing over-fitting; well-measured antigens remain more stable while allowing antigens with fewer measurements greater freedom of movement. The motion of a particle during a time step follows Newton’s second law of motion: $a_{i}=\frac{F_{i}}{m_{i}}$, where $a_{i}$ denotes the acceleration of particle *i*.

A critical distinction between Topolow and force-directed graph layouts is in how distances are treated. In graph layouts, edges typically have uniform lengths and serve mainly to keep connected nodes close together (Fruchterman and Reingold 1991). In contrast, our model implements the true distances through the free lengths of springs, making it suitable for assessing quantitative relationships.

**Figure 1.**
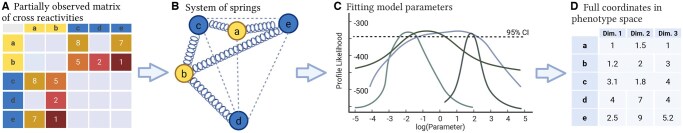
Schematic of the model. (A) Assay results are organized into a similarity matrix. Points a and b are reference and c, d, and e are test antigens. Measurements are possible only between opposite pairs. (B) The matrix is converted into a spring system, in which, antigens are represented as particles connected by springs wherever an assay measurement exists. Dashed lines are missing measurements to be estimated by Topolow. (C) Model parameters are fitted to maximize the likelihood of validation data. (D) Topolow finds the optimal dimensionality and coordinates of all test and reference antigens. Figure created in BioRender: https://BioRender.com/ap0pwr0.

The output provides optimal *N*-dimensional coordinates for all test and reference antigens. These coordinates can be used directly for distance calculations and downstream analyses, or passed to dimensionality reduction methods such as principal component analysis (PCA), *t*-distributed stochastic neighbor embedding (t-SNE), uniform manifold approximation and projection (UMAP), or other techniques. By default, Topolow visualizes high-dimensional output in 2D using PCA, with appropriate scaling to preserve linearity and interpretability of distances (see Fig. 1).

### 2.3 Optimization approach

The algorithm starts by setting initial positions for nodes and optimizes the coordinates to minimize the mean absolute error (MAE) between distances and observations. Rather than a uniformly random initialization, we employ a time-homogeneous Brownian-like Ornstein–Uhlenbeck diffusion process (Lande 1976) to specify the initial antigenic trait distribution (Bedford *et al.* 2014). The initialization affects only the starting positions and does not constrain the final solution, as particles move freely during the optimization process.

Optimization proceeds in the *N*-dimensional space by simulating the physical system’s dynamics. A random permutation of list of particles is created at the beginning of each cycle of simulations. At each time step, *t*, a single particle *i* is selected and its position is updated based on the pairwise interaction forces with one other particle, while all other particles remain fixed. We allow the forces to act on the particle and move it, starting from a stationary state, for one unit of time. Spring and repulsive forces and their induced displacements ($d_{s,i,t}$ and $d_{r,i,t}$) are calculated as follows:

For spring forces, assuming a constant force during a small displacement results in constant acceleration that can be calculated using Newton’s second law: $a_{i,t}=\frac{F_{s,i,t}}{m_{i}}$. The displacement of the particle during the time step, $d_{s,i,t}$, can be derived through the following independent relationships. First, the speed at the end of the time period is calculated as:

(3) $$V_{t+1}=V_{t}+a_{s,i,t}\Delta t.$$

Setting the time interval, Δt to 1 and initial velocity $V_{t}=0$ yields:

(4) $$a_{s,i,t}=V_{t+1}.$$

The average velocity in a motion with constant acceleration is the average of the velocities at the beginning and end of the motion:

(5) $$\bar{V}=\frac{V_{t}+V_{t+1}}{2}=\frac{V_{t+1}}{2}=\frac{a_{s,i,t}}{2};$$

Equation (4) was used at the last step. We can estimate $d_{s,i,t}$ using trapezium rule in discrete numerical integration as $d_{s,i,t}=\bar{V}\Delta t$. Setting Δt=1 and using (5) and (4) (in this order):

(6) $$d_{s,i,t}=\frac{a_{s,i,t}}{2}=\frac{V_{t+1}}{2}.$$

To express $d_{s,i,t}$ in terms of the latest distance of particles *i* and *j*, $r_{ij,t}$, and the constants of the model, we use the rule of conservation of energy:

(7) $$\frac{1}{2}kr_{ij,t}^{2}+\frac{1}{2}m_{i}V_{t}^{2}=\frac{1}{2}k{\left(r_{ij,t}-d_{s,i,t}\right)}^{2}+\frac{1}{2}m_{i}V_{t+1}^{2}.$$

Substituting $V_{t}=0$ and using (4) and (6), gives:

(8) $$d_{s,i,t}=\frac{2kr_{ij,t}}{4m_{i}+k}.$$

For repulsive forces, plugging $F_{r,ij,t}$ in Newton’s second law yields:

(9) $$\frac{c}{r_{ij,t}^{2}}=m_{i}a_{r,i,t}.$$

$F_{r,ij,t}$ is inversely proportional to $r_{ij,t}^{2}$, hence, it is comparably small. Consecutively, the resulting displacement $d_{r,i,t}$ is small compared to $r_{ij,t}$, and we can approximate by assuming a constant force during the motion. Then, similar to (6), we will have $d_{r,i,t}=\frac{a_{r,i,t}}{2}$. Plugging it in (9)
 

(10) $$d_{r,i,t}=\frac{c}{2m_{i}r_{ij,t}^{2}}.$$

To handle threshold constraints, spring forces are only activated when the current distance falls below the distance corresponding to the threshold value. The force works to push the distance closer to at least the threshold value. A similar scenario happens in the opposite direction for lower bound thresholds.

The loss function in Topolow is based on MAE:

(11) $$\text{Loss}(\theta )=\frac{1}{n}\sum_{D_{ij}\neq \text{NA}}|D_{ij}-r_{ij}|,$$

where θ represents the model parameters. At the end of each cycle (when the positions of all particles are updated), the loss is calculated and the convergence checked against a threshold. The full pseudo-code of the algorithm and some implementation notes are provided in the Supplementary Methods, available as supplementary data at *Bioinformatics* online.

### 2.4 Parameter fitting and likelihood analysis

There are four parameters in the model: the spring constant (*k*), the repulsion constant (*c*), the dimensionality of the antigenic space (*N*), and the cooling rate (α). An adaptive Monte Carlo (AMC) sampling approach (Bucher 1988) was employed to construct the likelihood surface for each dataset and determine the optimal value of each parameter, prior to using the algorithm for mapping. Full details are provided in the Supplementary Methods, available as supplementary data at *Bioinformatics* online.

Notably, likelihood-based determination of dimensionality enhances analytical precision compared to the *ad hoc* dimensionality optimization protocols adopted in previous studies (Smith *et al.* 2004, Bedford *et al.* 2014). Insufficient dimensionality introduces non-uniform distortions in pairwise distances, where some distances become artificially inflated while others are compressed (Fig. S1, available as supplementary data at *Bioinformatics* online). These distortions can generate spurious clusters or merge distinct antigenic groups, potentially confounding biological interpretation.

### 2.5 Antigenic velocity

To quantify the rate and direction of antigenic change, we introduce the antigenic velocity vector for each isolate *i*:

(12) $$v_{i}=\frac{\sum_{\begin{array}{c} j:\;t_{j}<t_{i}\\ j\in C(i) \end{array}}K_{ij}\frac{x_{i}-x_{j}}{t_{i}-t_{j}}}{\sum_{\begin{array}{c} j:\;t_{j}<t_{i}\\ j\in C(i) \end{array}}K_{ij}},$$

where $x_{k}$ and $t_{k}$ are the map coordinates and sampling year of isolate *k*. Background points *j* are restricted to the same clade C(i) when a phylogeny is supplied; otherwise, all earlier antigens are eligible. Each pair is weighted by a kernel $K_{ij}$:

(13) $$K_{ij}=\text{exp}\;\left(-\frac{∥x_{j}-x_{i}{∥}^{2}}{2\sigma_{x}^{2}}\right)\;\text{exp}\;\left(-\frac{{\left(t_{j}-t_{i}\right)}^{2}}{2\sigma_{t}^{2}}\right),$$

so that only temporally and antigenically proximate ancestors contribute appreciably. $\sigma_{x}$ and $\sigma_{t}$ are kernel bandwidths in antigenic and temporal dimensions. Bandwidth parameters and clades can be set by the user or calculated automatically based on the data. Bandwidth calculations, dynamic depth-based clade detection based on “average leaf-to-backbone distance”, and limitations of the approach are discussed in the Supplementary Methods, available as supplementary data at *Bioinformatics* online.

The magnitude of $v_{i}$ represents the antigenic change per unit of time (e.g. two-fold per year), and its orientation shows the direction of drift.

### 2.6 Simulation study design

To benchmark the relative performance of Topolow and MDS, we carried out a simulation study. Three essential features of antigenic evolution were incorporated in simulated data: directional selective pressure, clustered antigenic phenotypes, and measurement noise (Bush *et al.* 1999, Bedford *et al.* 2014). Arbitrary values were selected to generate these features. Dataset complexity was characterized by dimensionality, with antigenic coordinates generated in 2-, 5-, and 10-D spaces. A three-step process was implemented for coordinate generation. Initially, a trend vector was established to represent a directional selective pressure and antigenic drift observed in viral data (Bush *et al.* 1999), with a linear progression from −10 to 10 arbitrary units across the simulated antigenic space. Five distinct antigenic clusters were then positioned along this trend vector, with cluster centers drawn from a uniform random distribution over the trend length. Biological variation was subsequently introduced through two layers: local spread of antigens around cluster centers was drawn from a multidimensional normal distribution ($\sigma_{c}=1$), and global phenotypic randomness was implemented ($\sigma_{g}=3.3$).

For each dimensionality, 250 antigenic phenotypes were generated and split into 150 test and 100 reference antigens to mirror typical experimental conditions. For each dimensional scenario, three datasets with increasing proportions of missing measurements were created (70%, 85%, and 95%). Missing values followed a distance-dependent pattern, preferentially occurring between temporally and antigenically distant antigens. Then three variants for each scenario were developed: (i) “Original”—the base dataset; (ii) “+Noise”—original data with added Gaussian noise (μ=0, σ=5% of mean distances); and (iii) “+Noise+Bias”—original data with both random noise and a constant bias (5% of mean distances). Table S1, available as supplementary data at *Bioinformatics* online, shows a summary of the simulated datasets.

### 2.7 Cross-validation experimental setup

Model performance was evaluated through 20-fold cross-validation experiments on empirical and simulated data. Available measurements in each antigenic distance matrix were randomly partitioned into training (95%) and test (5%) sets. Model parameters were fitted to the training data using AMC simulation (see Supplementary Methods, available as supplementary data at *Bioinformatics* online), after which both Topolow and MDS models were employed to predict test measurements. Prediction accuracy was quantified using validation MAE for within-data comparisons and validation mean absolute percentage error (MAPE) for cross-data evaluations between predicted and test antigenic distances.

### 2.8 Implementation

Topolow is implemented as an open-source R package (requires *R*≥4.3.2) and is freely accessible on CRAN (Arhami and Rohani 2025). Optimization of parameters within 5% error tolerance for the H3N2 HI dataset requires 40 initial and 100 AMC samples, completing in approximately 20 min on an M1 Mac (3.2 GHz, 20 cores, 32 GB RAM). Subsequent antigenic map generation requires only 5 s on identical hardware. For larger datasets, the algorithm supports parallel processing through distributed computing frameworks to further reduce computation time.

## 3 Results

### 3.1 Validation on simulated data

To rigorously benchmark the performance of Topolow, we first designed a comprehensive simulation framework (see Section 2 and Fig. S2, available as supplementary data at *Bioinformatics* online).

#### 3.1.1 Performance comparison with MDS

**Figure 2.**
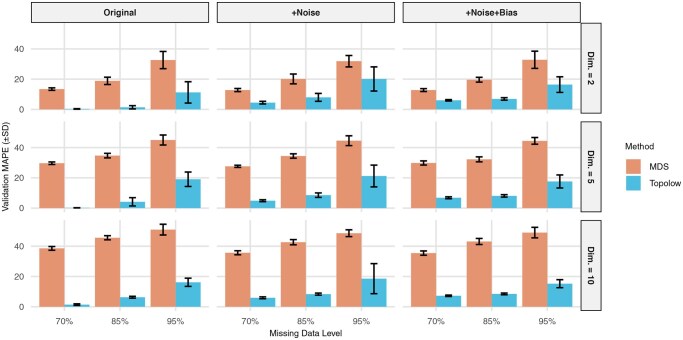
Quantification of model performance on simulated data. Validation MAPE was compared across data generated in dimensions 2, 5, and 10, three variants of data: original, +Noise (original distances plus a 5% random noise), +Noise+Bias (original distances plus a 5% random noise and a 5% bias), and three missing percentages: 70%, 85%, and 95%. Optimal dimensionality of each method for each scenario was determined beforehand (Fig. S3 and Table S2, available as supplementary data at *Bioinformatics* online). Numerical results are reported in Table S3, available as supplementary data at *Bioinformatics* online.

Topolow was evaluated against the commonly used MDS method for antigenic mapping, as implemented in RACMACS (Wilks 2024). Among existing approaches (e.g. Bedford *et al.* 2014, Han *et al.* 2019), Topolow and traditional MDS were distinguished by their function as standalone tools for antigenic characterization without requiring additional data types. As demonstrated in Fig. 2, both methods exhibited decreased accuracy with increasing data complexity (dimensionality) and sparsity. Notably, Topolow consistently achieved significantly lower MAPE than MDS—typically multiple orders of magnitude smaller—across all scenarios (paired *t*-tests calculated in Tables S4 and S5, available as supplementary data at *Bioinformatics* online, *P* < .0001 for all scenarios). Visual inspection of maps created by Topolow and MDS also shows that while MDS produces distorted relationships and fails to position all points, Topolow maintains the clear cluster structure almost identical to the original data. Figure S4, available as supplementary data at *Bioinformatics* online, depicts the comparison for the most challenging scenario.

#### 3.1.2 Completeness of antigenic maps

A critical feature of Topolow is its complete positioning of antigens in maps regardless of dimensionality or missing data. In contrast, the completeness of MDS output deteriorates as both dimensionality and the proportion of missing data increase (Table S6, available as supplementary data at *Bioinformatics* online).

#### 3.1.3 Bias analysis

The distributions of error for MDS maps exhibited positive bias across all scenarios, while Topolow consistently achieved near-zero biases (Table S7, available as supplementary data at *Bioinformatics* online). It is notable because antigenic distances are usually compared against a threshold and biases can shift antigens from non-novel to novel area, or vice versa.

#### 3.1.4 Robustness to input noise and bias

One of the potential advantages of creating an antigenic map is the reduction of errors by using multiple measurements to determine the position of each antigen. We test this hypothesis for each method by comparing the MAPE of its results with known input errors. The error metrics are defined as follows: (i) Input MAPE: The average percentage of absolute differences between distances in the noisy/biased and the original variants of each scenario. It is the baseline noise in the input data, representing the experimental and measurement errors. (ii) MDS MAPE: The MAPE between the distances on the maps created by MDS and the original distances. (iii) Topolow MAPE: The MAPE between the distances on the maps created by Topolow and the original distances.

Table 1 shows the comparison of MAPEs for 5D scenarios (other scenarios in Table S8, available as supplementary data at *Bioinformatics* online). Topolow consistently achieves lower MAPE than MDS and typically the baseline. This phenomenon can be attributed to Topolow’s network-based noise cancelation mechanism. When inconsistencies arise from random measurement errors, the spring-based physical model naturally mitigates them through competing forces within the network. This intrinsic error-dampening property demonstrates Topolow’s robust ability to reduce measurement noise in the data and reveal the underlying antigenic relationships.

**Table 1.** Comparison of MAPE (%) in the inputs due to added noise/bias against MAPE of locations determined by MDS and Topolow.^a^

Missing	Variant	Input	MDS	Topolow
70%	+Noise	5.693	29.917	3.205
70%	+Noise+Bias	8.488	29.769	6.507
85%	+Noise	5.420	37.041	4.073
85%	+Noise+Bias	7.867	35.523	6.328
95%	+Noise	4.791	48.159	5.459
95%	+Noise+Bias	7.173	47.703	7.324

a All errors are calculated against the known non-noisy ground truth. Only data generated with 5 dimensions is shown; see full data in Table S8, available as supplementary data at *Bioinformatics* online.

### 3.2 Application to empirical datasets

We evaluated Topolow using three distinct empirical datasets that represent different challenges in antigenic cartography. The first is the extensively studied dataset of HI assays of H3N2 influenza antigens from Smith *et al.* (2004), which serves as the gold standard due to its careful curation and extensive validation. The second is a larger, uncurated, HIV-1 neutralization dataset from Los Alamos National Labs (Yoon *et al.* 2015), which presents additional challenges of potential noise and the absence of clear established antigenic patterns. Lastly, the smaller dataset of dengue virus (DENV) neutralization titers (Katzelnick *et al.* 2015), which is unique in comprising four distinct serotypes of DENV.

#### 3.2.1 H3N2 influenza analysis (1968–2003)

The H3N2 HI dataset (Smith *et al.* 2004) contains 4215 measurements between 273 antigens (test) and 79 antisera (reference), spanning 1968–2003. This represents 20% of all possible test-reference combinations, resulting in 91% missing values in a symmetric matrix of all antigens and antisera.

**Figure 3.**
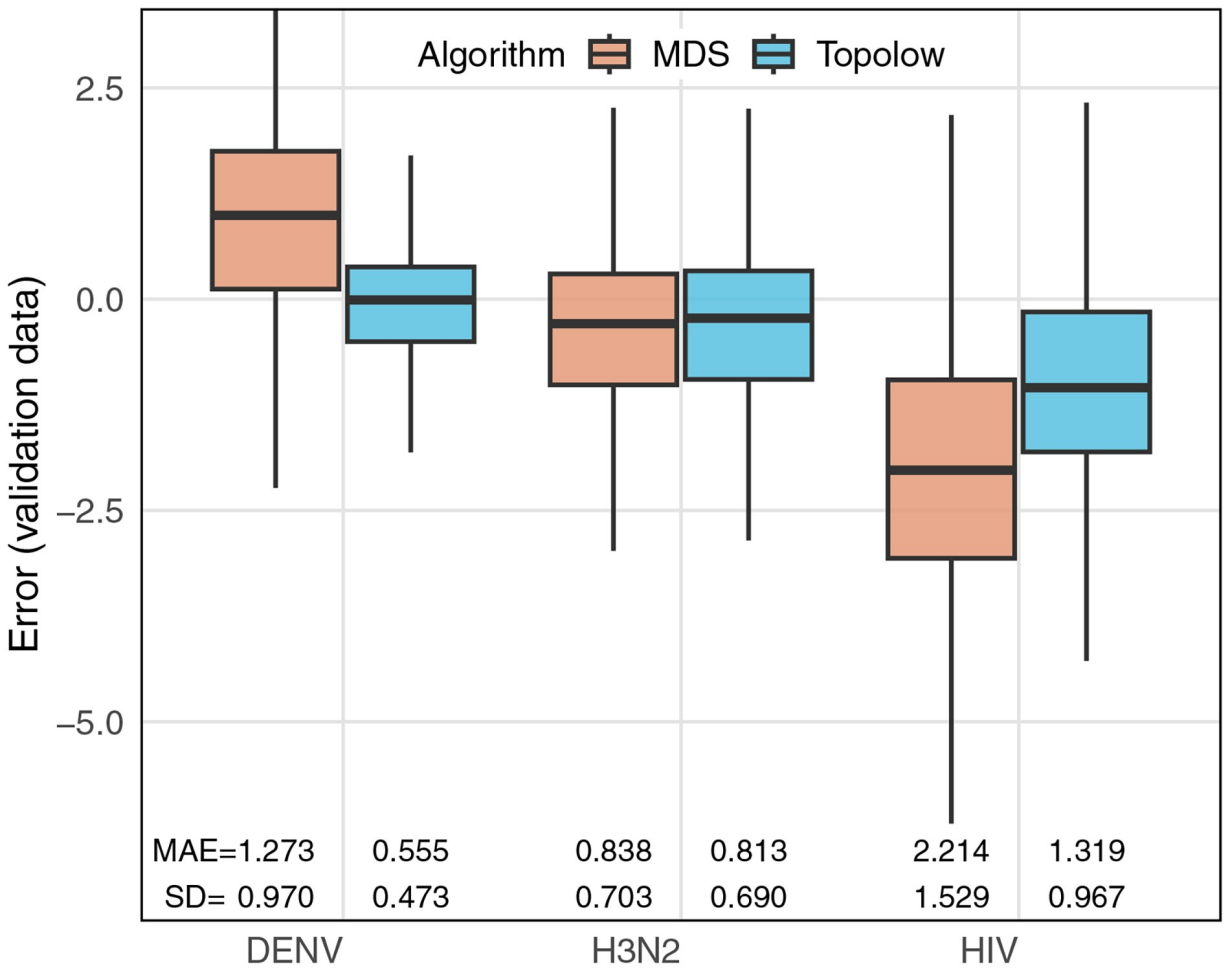
The distribution, MAE, and SD of validation errors across methods, for antigenic distances of dengue viruses with 5th to 95th percentile (0–5.858), H3N2 viruses with 5th–95th percentile (0–7.665) and $\log(IC_{50})$ values for HIV with 5th–95th percentile (0–3.864).

Topolow’s likelihood analysis and elbow method for MDS identified the data to be 5D and 4D, respectively (Figs. S5 and S6A, available as supplementary data at *Bioinformatics* online). Topolow achieved a validation MAE of 0.81±0.69 $log_{2}$ units, comparable with MDS (MAE = 0.84±0.70) although we note that in 4D, MDS failed to find the location of all antigens (Table S6, available as supplementary data at *Bioinformatics* online). Figure 3 shows the distribution of validation errors and detailed performance metrics across methods.

**Figure 4.**
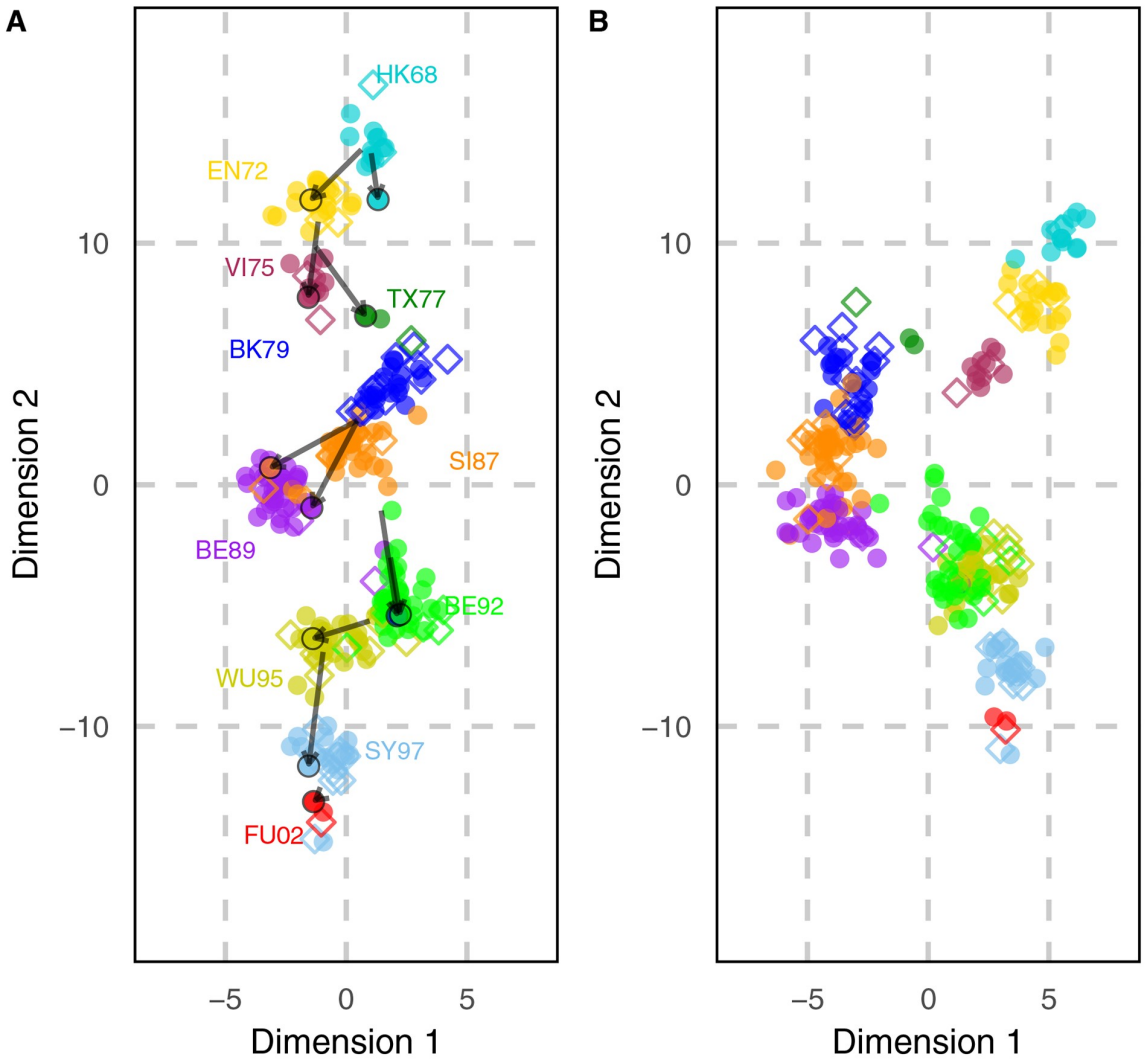
Comparison of antigenic maps estimated by (A) Topolow and (B) MDS for H3N2 HI titers. Test antigens are shown as colored circles and reference antigens as colored diamonds, with colors denoting antigens’ clusters inferred by Smith *et al.* (2004). The largest antigenic velocity vector for each cluster is shown on map A. The map showing all vectors is provided in Fig. S7, available as supplementary data at *Bioinformatics* online. Each unit on the map corresponds to a two-fold change in HI titer.

The 2D projection of the antigenic map generated by Topolow (Fig. 4A) is strikingly similar to the map published by Smith *et al.* (2004) using MDS, with consistently identified key antigenic clusters. The presented antigenic velocities are limited to the largest vector per cluster. The resulting arrows closely correspond to the flagship reverse-genetics antigenic mapping work of Koel *et al.* (2013) in terms of arrows’ lengths and details—e.g. antigenic velocity vectors of BE92 cluster orient out of SI87, not the temporally closer BE89—with the potential advantage that our vectors are calculated automatically, mark the exact shifted strain, needing only the sole new isolate, rather than requiring a retrospective consensus virus for the new cluster (Koel *et al.* 2013).

**Figure 5.**
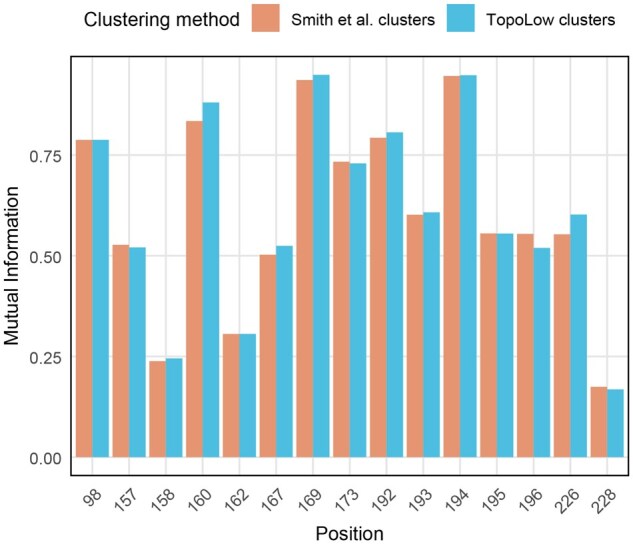
Evaluation of discriminatory power of amino acid substitutions in antigenic and receptor binding sites. Only positions with MI > 0.05 are shown. In total, ∑(MI in Topolow clusters) > ∑(MI in Smith *et al.* clusters) by 0.1.

K-means clustering of Topolow’s map (Fig. S8, available as supplementary data at *Bioinformatics* online) reveals a few differences in cluster assignments compared to those in Smith *et al.* (2004). To evaluate biological relevance, we quantified the discriminatory power of amino acid substitutions at antigenic and receptor binding sites (Harvey *et al.* 2023) between both clustering schemes using mutual information (MI). Analysis demonstrates that Topolow’s clustering correlates strongly with these established immunologically significant positions (Fig. 5). The MI between Topolow’s clusters and key amino acid positions were typically equal or greater than that of Smith *et al.* (2004) clusters, indicating our purely data-driven approach captures antigenic clusters with equivalent or superior biological relevance.

#### 3.2.2 HIV and DENV neutralization data

The HIV neutralization dataset comprises $IC_{50}$ measurements between 284 antigens and 51 antibodies from HIV-1 subtypes B and C, the subtypes accounting for over 50% of global HIV infections (Buonaguro *et al.* 2007). The distance matrix is 94% incomplete.

$IC_{50}$ values directly measure antigenic dissimilarity but showed strong right-skew, necessitating log-transformation during pre-processing. Both Topolow and MDS identified 2 as the optimal dimensionality for mapping these data (Figs. S9 and S6B, available as supplementary data at *Bioinformatics* online). Topolow achieved a validation MAE of 1.32±0.97 $log_{2}$ units—a 41% improvement over MDS (MAE = 2.21±1.53).

**Figure 6.**
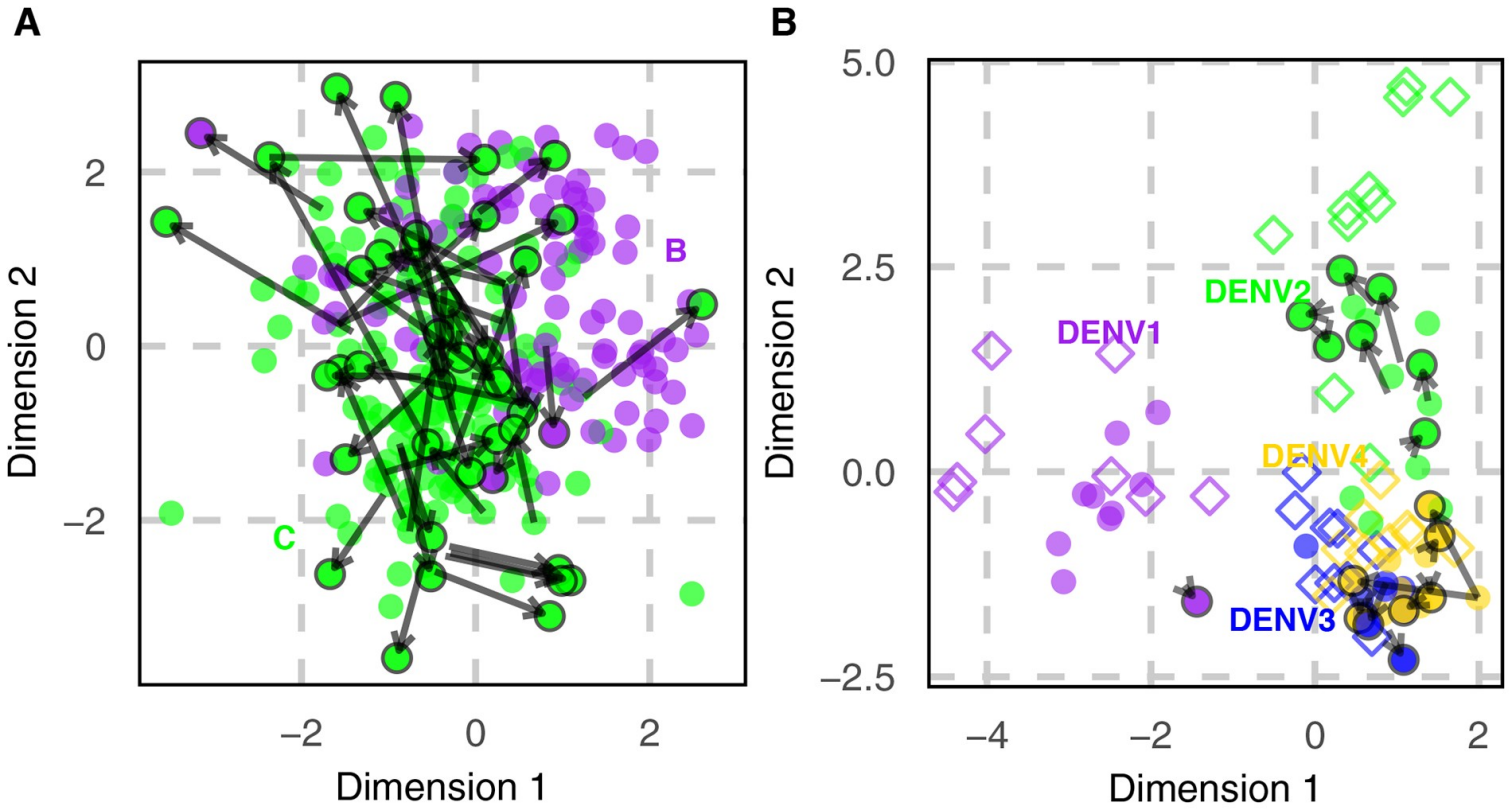
(A) 2D visualization of HIV antigenic maps created by Topolow, colored by subtypes. Antigenic velocity arrows longer than 1 unit are shown. (B) Same map with DENV data. Arrows longer than 0.1 unit are shown. Each unit on the maps corresponds to a two-fold change in titer. See maps showing all arrows in Figs. S10 and S12, available as supplementary data at *Bioinformatics* online, and similar maps by MDS in Figs. S13 and S14, available as supplementary data at *Bioinformatics* online.

The resulting antigenic map (Fig. 6A) reveals a pattern of antigenic clustering by viral subtype. It should be noted that antigenic drift in HIV does not exhibit a systematic trend, and larger velocity vectors are common in subtype C, corroborating previous findings of the highest genomic substitution rate in this subtype (Patino-Galindo and Gonzalez-Candelas 2017).

The DENV data comprises 47 viruses and 1839 neutralization titers. The distance matrix is 77% incomplete. Topolow selected 10 as the optimal dimensionality and after positioning every virus achieved a validation MAE of 0.56±0.68 ${\;\text{log}\;}_{2}$ units, 56% lower than MDS (2.21±1.53) in 3D (optimal dimensions). The resulting map (Fig. 6B) preserves the expected serotype structure while demonstrating slightly smaller distances compared to the map produced by MDS (Katzelnick *et al.* 2015). Comparison with the data proves that the difference is due to a bias of +1 fold in MDS results (Fig. 3). Consistent with being the most prevalent serotype for decades (Costa *et al.* 2012), DENV2 demonstrates a more pronounced trend in its antigenic evolution, with arrows indicating evolution away from other serotypes. More detailed analyses on HIV and DENV antigenic maps are warranted, but beyond the scope of this article.

#### 3.2.3 Convergence stability analysis

It is critical for policy applications that antigenic characterizations be consistent across multiple runs of any method. To quantify the stability of Topolow and MDS, 15 independent 2D maps were created by each method for all three empirical datasets to have 105 paired maps ($\left(\begin{array}{c} 15\\ 2 \end{array}\right)=105$) for each of the six method-data combinations, yielding sufficient power in paired *t*-tests. MDS was allowed to repeat each run 1000 times and keep the result with the smallest sum of squared errors to avoid convergence to a local optimum. Run-to-run variation for each method-data combination was evaluated by calculating Procrustes sum of squared errors (PSSE) for the 105 paired maps. Since each pair of maps is generated by the same method on the same data, they should not, in principle, differ significantly.

The mean and variance of PSSEs are shown in Table 2. Topolow demonstrated better stability on H3N2 and HIV—larger data with higher missing proportion—with mean PSSE orders of magnitude smaller than MDS for both datasets, confirmed by paired *t*-tests (*P* < .001). However, the mean PSSE of MDS was significantly smaller on DENV—small data with lower missing proportion. The standard deviations of PSSEs were lower for Topolow in all cases, indicating more consistent performance across runs, confirmed by *F*-tests for variances (*P* < .001).

**Table 2.** Run-to-run stability analysis (Procrustes sum of squares).

Data	Method	Mean	SD	*t*-test
H3N2	TopoLow	37.70	22.35	*P* < .001
H3N2	MDS	3665.55	3121.34	
HIV	TopoLow	483.88	244.48	*P* < .001
HIV	MDS	1115.81	339.23	
DENV	TopoLow	61.85	23.13	*P* < .001
DENV	MDS	43.22	37.74	

## 4 Discussion

Understanding and quantifying the antigenic evolution of rapidly evolving viral pathogens, including influenza (Smith *et al.* 2004), SARS-CoV-2 (Wilks *et al.* 2023), HIV (Haynes and Montefiori 2006), dengue (Katzelnick *et al.* 2015), and hepatitis C (Lara *et al.* 2008), is crucial for public health surveillance and the design of effective intervention strategies (Hadfield *et al.* 2018). Current methods for antigenic cartography, primarily based on MDS, face limitations when handling complexity and sparse datasets—a common issue as experimental data grow in size. Up to 95% of possible measurements may be missing in large-scale studies, compromising the accuracy and stability of resulting maps (Haynes and Montefiori 2006, Wikramaratna *et al.* 2013).

We have developed Topolow, a physics-inspired optimization framework that effectively addresses key challenges in antigenic cartography. When tested on various empirical and simulated datasets, Topolow demonstrated several advantages over traditional MDS approaches: (1) Superior handling of missing data through independent optimization of pairwise relationships. (2) Automatic determination of optimal dimensionality through likelihood-based estimation. (3) Orders of magnitude better consistency of results across multiple runs. (4) Effective reduction of experimental noise and bias.

The mechanistic foundations of these improvements derive from several key algorithmic features. First, while traditional MDS approaches calculate global gradient vectors that are susceptible to local optima, Topolow’s sequential pairwise optimization approach updates particle positions one pair at a time. This reduces susceptibility to local minima and decreases sensitivity to missing data patterns, as each subsequent pair can help the system escape suboptimal configurations. Second, the physics-inspired spring network organically distributes and dampens individual disturbances—when noisy measurements attempt to pull a particle to an incorrect position collective forces from other connections resist this deviation, averaging out errors. Third, Topolow’s stability improvements are further enhanced by its robust error modeling using Laplace distribution, providing better resilience against outliers in serological data. Fourth, the cooling schedule creates a balanced exploration–exploitation approach, allowing initial broad exploration followed by gradual refinement. Finally, continuous stochasticity through random pair selection in each iteration introduces persistent randomization throughout optimization, helping the algorithm thoroughly explore the solution space and avoid premature convergence to suboptimal configurations. These mechanisms work synergistically to overcome the limitations of gradient-based approaches when handling sparse datasets, reduce sensitivity to experimental noise, and produce more consistent maps.

The smaller improvement observed with empirical data compared to our simulation studies reflects complex non-metric characteristics in biological reality compared to synthetic data. Furthermore, the H3N2 dataset represents a carefully curated benchmark that has been extensively analysed with existing RACMACS implementation of MDS, potentially approaching optimal achievable performance. This underscores the importance of evaluating algorithmic performance across diverse datasets with varying characteristics, as demonstrated by the substantially larger improvements (56% and 41%) achieved with DENV and HIV neutralization data.

We have introduced the new concept of antigenic velocity, which offers a potentially insightful view of the direction and magnitude of evolution of isolates through antigenic space relative to the immunity landscape created by their immediate predecessors. It indicates whether the virus has become relatively better at escaping neutralization by certain sera versus others. The antigenic velocity field reveals punctuated shifts in H3N2 (Fig. 4A and Fig. S7, available as supplementary data at *Bioinformatics* online), scattered movement in HIV-1 (Fig. 6A), and an evolutionary trend away from other serotypes in DENV2 (Fig. 6B). One large vector, or several small aligned vectors, has typically preceded the emergence of new antigenic clusters in influenza, suggesting that velocity hotspots may offer early warning of future lineage replacements.

By uncovering distances between all antigens, including those lacking direct measurements, Topolow effectively multiplies the training data available for downstream machine learning models (e.g. Lara *et al.* 2008, Lees *et al.* 2010, Steinbruck *et al.* 2014, Jia *et al.* 2024). The method can characterize any continuous and relational phenotype under directional selection pressure, extending its utility beyond viral antigenic mapping to broader evolutionary studies (Pybus and Rambaut 2009).

Recent work has demonstrated the value of combining antigenic characterization with other data types for surveillance and vaccine strain selection (Liao *et al.* 2008, Xia *et al.* 2009, Steinbruck *et al.* 2014, Ritchie *et al.* 2015, Huang *et al.* 2017, Harvey *et al.* 2023). Topolow could enhance such efforts by providing comprehensive, accurate, and stable antigenic characterization across the viral strains. Topolow’s ability to predict antigenic phenotypes for under-characterized strains could be particularly valuable for early detection of emerging variants and examining early indicators of cluster success (Neher *et al.* 2016).

Limitations of the current implementation include: (i) inability to connect completely disconnected subgraphs in the measurement network, (ii) assumption of directional selection pressure in temporal initialization, and (iii) relative computational intensity.

## Supplementary Material

btaf372_Supplementary_Data

## Data Availability

The data underlying this article are available in Zenodo, at https://doi.org/10.5281/zenodo.15620983

## References

[btaf372-B1] Arhami O , Rohani P. Topolow R package version 1.0.0. 2025.

[btaf372-B2] Bedford T , Suchard M, Lemey P et al Integrating influenza antigenic dynamics with molecular evolution. eLife 2014;3:e01914. 24497547 10.7554/eLife.01914 PMC3909918

[btaf372-B3] Bravo M. A simulation to evaluate the ability of nonmetric multidimensional scaling to recover the underlying structure of data under conditions of error, method of selection, and percent of missing pairs. Ph.D. thesis, The University of Texas at Austin, 2002.

[btaf372-B4] Bucher CG. Adaptive sampling—an iterative fast Monte Carlo procedure. Struct Saf 1988;5:119–26.

[btaf372-B5] Buonaguro L , Tornesello ML, Buonaguro FM. Human immunodeficiency virus type 1 subtype distribution in the worldwide epidemic: pathogenetic and therapeutic implications. J Virol 2007;81:10209–19. 17634242 10.1128/JVI.00872-07 PMC2045484

[btaf372-B6] Bush R , Bender C, Subbarao K et al Predicting the evolution of human influenza A. Science 1999;286:1921–5. 10583948 10.1126/science.286.5446.1921

[btaf372-B7] Cai Z , Zhang T, Wan X. A computational framework for influenza antigenic cartography. PLoS Comput Biol 2010;6:e1000949. 20949097 10.1371/journal.pcbi.1000949 PMC2951339

[btaf372-B8] Costa R , Voloch C, Schrago C. Comparative evolutionary epidemiology of dengue virus serotypes. Infect Genet Evol 2012;12:309–14. 22226705 10.1016/j.meegid.2011.12.011

[btaf372-B9] Fruchterman T , Reingold E. Graph drawing by force-directed placement. Softw: Pract Exp 1991;21:1129–64.

[btaf372-B10] Hadfield J , Megill C, Bell SM et al Nextstrain: real-time tracking of pathogen evolution. Bioinformatics 2018;34:4121–3. 29790939 10.1093/bioinformatics/bty407 PMC6247931

[btaf372-B11] Han L , Li L, Wen F et al Graph-guided multi-task sparse learning model: a method for identifying antigenic variants of influenza A(H3N2) virus. Bioinformatics 2019;35:77–87. 29878046 10.1093/bioinformatics/bty457 PMC6298058

[btaf372-B12] Harvey W , Benton D, Gregory V et al Identification of low- and high-impact hemagglutinin amino acid substitutions that drive antigenic drift of influenza A(H1N1) viruses. PLoS Pathog 2016;12:e1005526. 27057693 10.1371/journal.ppat.1005526 PMC4825936

[btaf372-B13] Harvey W , Davies V, Daniels R et al A Bayesian approach to incorporate structural data into the mapping of genotype to antigenic phenotype of influenza A(H3N2) viruses. PLoS Comput Biol 2023;19:e1010885. 36972311 10.1371/journal.pcbi.1010885 PMC10079231

[btaf372-B14] Haynes B , Montefiori D. Aiming to induce broadly reactive neutralizing antibody responses with HIV-1 vaccine candidates. Expert Rev Vaccines 2006;5:347–63. 16827619 10.1586/14760584.5.3.347 PMC2716009

[btaf372-B15] Hensley S , Das S, Bailey A et al Hemagglutinin receptor binding avidity drives influenza a virus antigenic drift. Science 2009;326:734–6. 19900932 10.1126/science.1178258 PMC2784927

[btaf372-B16] Hirst G. Studies of antigenic differences among strains of influenza a by means of red cell agglutination. J Exp Med 1943;78:407–23. 19871338 10.1084/jem.78.5.407 PMC2135416

[btaf372-B17] Huang L , Li X, Guo P et al Matrix completion with side information and its applications in predicting the antigenicity of influenza viruses. Bioinformatics 2017;33:3195–201. 28637337 10.1093/bioinformatics/btx390

[btaf372-B18] Jia Q , Xia Y, Dong F et al MetaFluAD: meta-learning for predicting antigenic distances among influenza viruses. Brief Bioinform 2024;25:bbae395. 39129362 10.1093/bib/bbae395 PMC11317534

[btaf372-B19] Katzelnick LC , Fonville JM, Gromowski GD et al Dengue viruses cluster antigenically but not as discrete serotypes. Science 2015;349:1338–43. 26383952 10.1126/science.aac5017 PMC4876809

[btaf372-B20] Kobourov S. Spring embedders and force directed graph drawing algorithms. arXiv, arXiv:1201.3011, 2012 , preprint: not peer reviewed.

[btaf372-B21] Koel BF , Burke DF, Bestebroer TM et al Substitutions near the receptor binding site determine major antigenic change during influenza virus evolution. Science 2013;342:976–9. 24264991 10.1126/science.1244730

[btaf372-B22] Lande R. Natural selection and random genetic drift in phenotypic evolution. Evolution 1976;30:314–34. 28563044 10.1111/j.1558-5646.1976.tb00911.x

[btaf372-B23] Lapedes A , Farber R. The geometry of shape space: application to influenza. J Theor Biol 2001;212:57–69. 11527445 10.1006/jtbi.2001.2347

[btaf372-B24] Lara J , Wohlhueter R, Dimitrova Z et al Artificial neural network for prediction of antigenic activity for a major conformational epitope in the hepatitis C virus NS3 protein. Bioinformatics 2008;24:1858–64. 18628290 10.1093/bioinformatics/btn339

[btaf372-B25] Lees WD , Moss DS, Shepherd AJ. A computational analysis of the antigenic properties of haemagglutinin in influenza A H3N2. Bioinformatics 2010;26:1403–8. 20388627 10.1093/bioinformatics/btq160 PMC2913667

[btaf372-B26] Liao Y , Lee M, Ko C et al Bioinformatics models for predicting antigenic variants of influenza a/H3N2 virus. Bioinformatics 2008;24:505–12. 18187440 10.1093/bioinformatics/btm638

[btaf372-B27] Neher R , Bedford T, Daniels R et al Prediction, dynamics, and visualization of antigenic phenotypes of seasonal influenza viruses. Proc Natl Acad Sci U S A 2016;113:E1701–9. 26951657 10.1073/pnas.1525578113 PMC4812706

[btaf372-B28] Patino-Galindo JA , Gonzalez-Candelas F. The substitution rate of HIV-1 subtypes: a genomic approach. Virus Evol 2017;3:vex029. 29942652 10.1093/ve/vex029 PMC6007745

[btaf372-B29] Pybus O , Rambaut A. Evolutionary analysis of the dynamics of viral infectious disease. Nat Rev Genet 2009;10:540–50. 19564871 10.1038/nrg2583 PMC7097015

[btaf372-B30] Ritchie M , Holzinger E, Li R et al Methods of integrating data to uncover genotype–phenotype interactions. Nat Rev Genet 2015;16:85–97. 25582081 10.1038/nrg3868

[btaf372-B31] Smith D , Lapedes A, de Jong J et al Mapping the antigenic and genetic evolution of influenza virus. Science 2004;305:371–6. 15218094 10.1126/science.1097211

[btaf372-B32] Steinbruck L , Klingen T, McHardy A. Computational prediction of vaccine strains for human influenza A (H3N2) viruses. J Virol 2014;88:12123–32. 25122778 10.1128/JVI.01861-14 PMC4178748

[btaf372-B33] Wikramaratna P , Sandeman M, Recker M et al The antigenic evolution of influenza: drift or thrift? Philos Trans R Soc Lond B Biol Sci 2013;368:20120200. 23382423 10.1098/rstb.2012.0200 PMC3678325

[btaf372-B34] Wilks S. Racmacs: Antigenic Cartography Macros. R package version 1.2.9. 2024.

[btaf372-B35] Wilks SH , Mühlemann B, Shen X et al Mapping SARS-CoV-2 antigenic relationships and serological responses. Science 2023;382:eadj0070. 37797027 10.1126/science.adj0070 PMC12145880

[btaf372-B36] Xia Z , Jin G, Zhu J et al Using a mutual information-based site transition network to map the genetic evolution of influenza a/H3N2 virus. Bioinformatics 2009;25:2309–17. 19706746 10.1093/bioinformatics/btp423

[btaf372-B37] Yoon H , Macke J, West A et al CATNAP: a tool to compile, analyze and tally neutralizing antibody panels. Nucleic Acids Res 2015;43:W213–9. 26044712 10.1093/nar/gkv404 PMC4489231

[btaf372-B38] Zacour M , Ward B, Brewer A et al; Public Health Agency of Canada and Canadian Institutes of Health Influenza Research Network (PCIRN). Standardization of hemagglutination inhibition assay for influenza serology allows for high reproducibility between laboratories. Clin Vacc Immunol 2016;23:236–42. 10.1128/CVI.00613-15 PMC4783428 26818953

