# Supplementary material for: Topolow: a mapping algorithm for antigenic cross-reactivity and binding affinity assays
Source: Bioinformatics. 2025 Jun 25;41(7):btaf372. doi: 10.1093/bioinformatics/btaf372 (PMC12237505; doi:10.1093/bioinformatics/btaf372)
Supplement: btaf372_Supplementary_Data [file btaf372_supplementary_data.zip › Topolow_Bioinformatics_major_rev_2__submissionCopy_ (7) [SI]-1.pdf]

Supplementary Methods

The impact of missing values

Let D be the matrix of distances of all test and reference antigens constructed from the available titers. For illustration, a 4×4 matrix may take the form:

$$\mathrm{D} = \begin{array}{c} \text{Ref. Ag 1} \\ \text{Ref. Ag 2} \\ \text{Test Ag 1} \\ \text{Test Ag 2} \end{array} \overset{\begin{array}{cccc} \text{Ref. Ag 1} & \text{Ref. Ag 2} & \text{Test Ag 1} & \text{Test Ag 2} \end{array}}{\begin{pmatrix} 0 & D_{12} & - & - \\ D_{21} & 0 & D_{23} & - \\ - & D_{32} & 0 & D_{34} \\ - & - & D_{43} & 0 \end{pmatrix}}$$

In this hypothetical example, there are 4 points, 3 measurements, and 6 missing entries. To identify the coordinates of the 4 points, MDS, specifically metric MDS, specifies an objective function $S(X)$:

$$S(X) = \sum_{i<j \& D_{ij} exists} \left(D_{ij} - \|x_i - x_j\|\right)^2 . \tag{S1}$$

where x_i and x_j are the locations of antigens i and j on the generated map. Typically, Gradient Descent or Newton's method is used to find the minimum for $S(X)$. Both algorithms are guided towards the solution by partial derivatives of the objective function. The partial derivative with respect to the k-th coordinate of point i is:

$$\frac{\partial S}{\partial x_{ik}} = 2 \sum_{j \neq i \& D_{ij} exists} \left(\|x_i - x_j\| - D_{ij}\right) \frac{x_{ik} - x_{jk}}{\|x_i - x_j\|} . \tag{S2}$$

Notice that the formula for partial derivatives skips all the terms where D_{ij} is missing from the summation. Therefore, the accuracy of magnitude and direction of the gradient vector $\frac{\partial \vec{S}}{\partial x_i}$ is affected by missing elements in D. On the other hand, Topolow performs pairwise distance optimizations sequentially for each pair of nodes, the optimization direction is simply the vector connecting them. By avoiding the need to compute a gradient vector, Topolow has the potential to operate robustly even when a substantial portion of entries in D are missing.

Algorithm and implementation notes

We employ a cooling schedule where both spring stiffness k and repulsion strength c decrease according to a multiplicative function of iteration number. Specifically, at iteration t: $k(t) = k * (1-\alpha)^t$ and $c(t) = c * (1-\alpha)^t$ where α controls the cooling rate. This prevents oscillation and allows fine-scale adjustments in the final stages. The optimization terminates when the relative change in mean absolute error (MAE) between observed and projected antigenic distances falls below a preset threshold ϵ: $|(MAE_t - MAE_{t-1})|/MAE_t < \epsilon$.

Due to random initialization, the absolute positions and orientations of mapped points may vary between runs. However, the relative distances and spatial relationships between points remain consistent across different executions of the algorithm, producing visually similar maps.

Topolow is optimized to automatically detect the environment, submit parallel jobs on a SLURM cluster, and use multiple cores on an individual multi-core computer.

Algorithm 1 TopoLow (Topological Optimization for Low-Dimensional Mapping)

Require:
D: $n \times n$ distance matrix
k_0: initial spring constant
α: decay rate per iteration
c: repulsion constant
N: target dimensionality
ϵ: convergence threshold
Output:
X: $n \times N$ matrix of point coordinates in N-dimensional space
Initialize:
$X \leftarrow n \times N$ ▷ matrix of initial coordinates
$k \leftarrow k_0$ ▷ Current springs constant
degrees $\leftarrow$ array of node degrees from D
Main:
for each pair of points (i, j) where $i < j$ **do**
  $\delta \leftarrow X_j - X_i$ ▷ Vector between points
  $r \leftarrow \|\delta\|$ ▷ Current distance
  if $D_{i,j}$ is a measurement **then**
    if $D_{i,j}$ is thresholded **then**
      if threshold condition not met **then**
        $F \leftarrow (c/r^2)(\delta/r)$ ▷ Repulsive force
      end if
    else
      $F \leftarrow k(D_{i,j} - r)(\delta/r)$ ▷ Spring force
    end if
    $X_i \leftarrow X_i - 2F/(4\text{degrees}_i + k)$
    $X_j \leftarrow X_j + 2F/(4\text{degrees}_j + k)$
  else
    $F \leftarrow (c/r^2)(\delta/r)$ ▷ Apply repulsive force for missing measurements
    $X_i \leftarrow X_i - F/(2\text{degrees}_i)$
    $X_j \leftarrow X_j + F/(2\text{degrees}_j)$
  end if
end for
Calculate MAE
if converged **then**
  break
end if
$k \leftarrow k(1 - \alpha)$ ▷ Decay spring constant
$c \leftarrow c(1 - \alpha)$ ▷ Decay repulsion constant

Likelihood analysis

The choice of error model plays a key role in antigenic mapping due to the heterogeneous nature of serological data. While it may be tempting to model HI titer measurement errors using a normal distribution given its common usage and mathematical convenience, a Laplace distribution appears more appropriate for this application. HI titer measurements exhibit variability from multiple sources, including differences between laboratories, date of collection, observers, and assay details. Additionally, reporting low or high measurements as a threshold value introduces large errors. The Laplace distribution's heavier tails and greater robustness to outliers make it particularly well-suited for handling these heterogeneous sources of error. The Laplace model also aligns naturally with our choice of using MAE rather than squared error measures. The rationale for this choice is that larger errors often correspond to measurements at the limits of detection, and we do not want our optimization algorithm to be driven mostly by more inaccurate observations.

The likelihood function under the Laplace model for errors of n measurements, denoted as e_i, is:

$$L(\theta|e_1..e_n) = \prod_{i=1}^{n} \frac{1}{2b} \exp\left(-\frac{|e_i - \mu|}{b}\right), \tag{S3}$$

where μ and b are location and scale parameters of the distribution. It can be shown that the maximum likelihood estimates (MLE) for μ and b are $\hat{\mu} = \text{median}(e)$ and $\hat{b} = \frac{1}{n}\sum_{i=1}^{n}|e_i - \hat{\mu}|$. Since Laplace is a symmetric distribution, the sample median can be estimated with the sample mean: $\hat{\mu} = \text{mean}(e)$. Taking the negative log-likelihood and plugging in the MLEs:

$$\begin{aligned} NLL(\theta|e_1..e_n) &= n\log(2b) + \frac{1}{b}\sum_{i=1}^{n}|e_i - \mu| \\ &= n\log(2b) + n \\ &= n\log(2\text{MAE}) + n. \end{aligned} \tag{S4}$$

Adaptive Monte Carlo sampling for Parameter estimation

Unlike standard Monte Carlo methods that sample uniformly from the parameter space, the adaptive approach modifies the sampling distribution based on previously observed results. The sampling probability is proportional to the joint distribution of NLL. Thus, to obtain each sample, we reconstruct the NLL surface in the space of all parameters using a kernel density estimator (KDE). This approach concentrates sampling in regions of the parameter space that are more likely to contain optimal values.

To evaluate the validation error with each set of parameters, we used k-fold cross-validation with $k = 20$. For each fold, we randomly removed 5% of measurements for testing. The likelihood of each set of parameters was also calculated as the sum of likelihoods on all validation folds.

To initialize the parameter search, we generate 50 parameter combinations using Latin hypercube sampling, ensuring broad and efficient coverage of the parameter space. The algorithm then draws additional AMC samples, updating the sampling distribution after each iteration. 100 is about the minimum number of AMC samples to get near-optimal errors for a dataset of about 200 antigens, however, 1000 runs or more is preferable. The peak of the final likelihood surface determines the optimal parameter values. Fig. S-5 and S-9 show profile likelihoods and 95% marginal confidence intervals for all parameters with H3N2 and HIV data sets. Although the 95% significance intervals found by the likelihood ratio test look tight, parameter sensitivity analyses in Fig. S-15 and S-16 show that MAE remains in the 5% neighborhood of the optimal MAE for a wide range of values of all parameters. In other words, model's performance is not too sensitive to the parameter values as long as the values are roughly in a neighborhood of the optimal values –$\sim$ 2 units in log scale for our H3N2 and HIV data.

Visual inspection

Figure S-4 shows the maps created by Topolow and MDS for our most challenging scenario (10 dimensions, 95% missing distances, with added noise and bias). This high quality of preservation of global structure is particularly noteworthy given the extreme sparsity and noise in this scenario.

Antigenic Velocity

Rationale

Antigenic velocity is intended to compare each focal antigen only with the samples that shaped the most relevant local evolutionary landscape, or recent population immunity in the case of fast-evolving viruses. Therefore, we use kernel functions to limit the effect of past antigens on velocity calculations of each antigen to its background. Equation 13 in the main text follows the classic kernel-smoother formalism (Wand and Jones 1995) with multivariate weighting in temporal and antigenic dimensions. We also augment it with an optional phylogenetically aware clade filter based solely on tree shape, so no rooting or molecular clock is required.

Kernel bandwidth selection

σ_{ag}, and σ_t are calculated automatically from the data with Silverman's rule-of-thumb (Silverman 1986) (implemented in function "bw.nrd" in "stats" package in R). They can also be overwritten by user as input arguments to the function. Obviously, the choice of bandwidths affects the velocity vectors by tightening or broadening the domain of background antigens (see the effects in Fig. S-17 to S-19). Therefore, they must be chosen carefully. Adjustability of bandwidths can be useful when there are established thresholds in the literature for temporal and antigenic differences, or the researcher has a specific research question (e.g. finding within-season evolution). Otherwise, the data-driven values found by Silverman's rule-of-thumb for kernel density estimation bandwidth are optimal as they are the bandwidth that minimizes the mean integrated squared error.

Temporal bandwidth:

$$\sigma_t = 1.06\,\hat{s}_{\Delta t}\,m^{-1/5} \quad \text{(Silverman's rule)}$$

where $\hat{s}_{\Delta t}$ is the standard deviation of time differences between all pairs of antigens in the data and $m = \binom{n}{2}$ is the count of all pairs.

For the H3N2 dataset $\sigma_t = 1.13$ years, so weights fall to 50% at a 1.75 year gap, compatible with serological findings in the literature (2-fold drop in immunity after 600 days (Petrie et al. 2015).)

Antigenic distance bandwidth:

Since vectors are based on locations in the 2D antigenic map, we calculate σ_x and σ_y –the bandwidths along the two map axes– and use their quadratic mean as the average scalar bandwidth.

$$\sigma_x = 1.06\,\hat{s}_x\,n^{-1/5}, \sigma_y = 1.06\,\hat{s}_y\,n^{-1/5},$$
$$\sigma_{ag} = \sqrt{\tfrac{1}{2}(\sigma_x^2 + \sigma_y^2)},$$

where $\hat{s}_x$ and $\hat{s}_y$ are the standard deviations of map coordinates along the each axis separately. This yields $\sigma_{ag} = 1.8$ map units ($\approx$ 3.5-fold) for H3N2, ensuring that close antigenic neighbors dominate K_{ij}.

Depth-based (phylogenetically-aware) clade definition

When a phylogeny is available we confine the background set $\mathcal{C}(i)$ to antigens that lie in the same clade as sample i. Because antigenic trees (i) are affected by choice of the root, (ii) often lack reliable molecular-clock branch lengths, and (iii) can be highly imbalanced (Gundersen and Vadstein 2024), we adopt a purely topological but data-driven procedure that:

– needs no rooting or branch-length calibration,

– scales automatically with the breadth of the sampled diversity, and
– uses a single intuitive parameter (clade depth) that the user may override if a different biological definition of "clade" is preferable.

We propose the "**Dynamic depth-based clade detection**" algorithm that finds local spread against the global backbone in 3 steps to define a novel branching metric.

1. **The backbone path**

   a. **Unit-length tree**. The supplied (possibly rooted) phylogeny is first unrooted and every edge is assigned length 1. This removes rate heterogeneity and temporal information; effect of time is handled explicitly by the kernel weight K_{ij} (Eq. 13).
   b. **Backbone (diameter) extraction**. We identify a diameter path—the longest shortest path between any two tips (Goddard and Oellermann 2011). Graph-theoretically, the diameter approximates the main axis of diversification across the sample.

2. **Average Leaf-to-Backbone Distance (ALBD)**
   Let d_ℓ be the shortest topological distance (in edges) from tip ℓ to the backbone. The set $\{d_\ell\}$ summarises how far leaves branch away from the diversification axis. We take the median of these values $\text{ALBD} = \text{median}\{d_\ell\}$ as a robust estimate of the typical side-branch depth (cf. Sackin's imbalance index (Sackin 1972)). ALBD is the clade-node depth cutoff; it is reported to the user and can be overridden.
3. **Assigning clades to tips**
   For each tip i:

   a. Count and walk ALBD edges towards the root (i.e. towards higher-order ancestors) to reach an internal node $v(i)$.
   b. Define $\mathcal{C}(i)$ as the set of all tips descended from $v(i)$.

Because every tip receives its own clade ancestor (internal node $v(i)$), overlapping clades are allowed; this ensures that an isolate is never compared with a lineage that split deeper than the chosen cutoff.

Relation to existing tree metrics

ALBD differs from the classic Sackin (Sackin 1972) and Colless (Colless 1982) indices (meant to quantify whole-tree imbalance); because it benchmarks local deviations against a global reference path.

It is also related to the "ladder length" of (Colijn and Gardy 2014) but allows for high-degree internal nodes and additionally, provides a numerical cutoff.

Practical considerations and limitations

Adjustability. Using a smaller depth threshold produces finer clades and therefore shorter velocity vectors, because the background is limited to very close relatives. Conversely, larger depths widen the background. For the H3N2 data, the data-driven ALBD was found to be 5, which is comparable to the mean distance between leaves and their clade roots in the tree produced by (Smith et al. 2004). Maps with antigenic velocity vectors for all data sets with a range of values for σ_t, σ_{ag}, and clade depth, including their data-driven values, are shown in Fig. S-17 to S-19.

Multiple diameter paths. If several backbones exist, we calculate $\{d_\ell\}$ for each, then take the mode of the entire values; this case is rare, and differences are minor.

Sampling bias. As with any tree metric, ALBD is affected by heavy under-sampling of certain lineages through its effect on tree topology. Users studying such datasets should supply an informed depth cutoff.

No branch-length information. Ignoring branch lengths avoids artefacts from rate variation and choice of phylogenetic model, but discards potentially informative signal. A length-weighted variant of ALBD is possible, but more involved.

Supplementary Figures and Tables

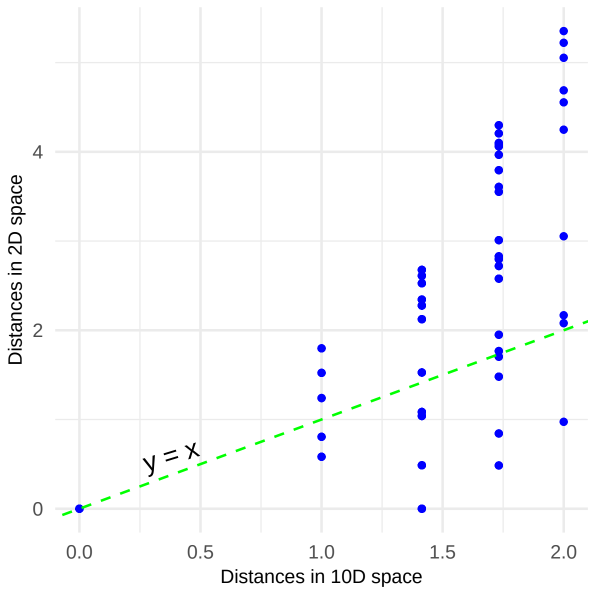

Fig. S-1. The pairwise distances in the original space (10D) versus the pairwise distances after reducing the dimensions to 2 using the MDS method applied to 15 points spread uniformly in a 10D space.

Given that the robust detection of clusters in antigenic evolution requires identification of the appropriate dimensionality, we propose a key innovation of Topolow is finding the optimal space dimensionality. By approaching the problem as a series of independent one-dimensional simulations –along the line connecting a pair of particles– Topolow circumvents the curse of dimensionality that plagues traditional methods. Unlike RACMACS (Wilks 2022), which requires calculating complex gradient vectors in high-dimensional spaces, Topolow only needs to consider movement along the direction connecting two nodes at each time, which allows it to maintain accuracy regardless of the overall dimensionality.

Table S-2. The optimal dimensionalities determined for MDS and Topolow for the simulated scenarios. Noting that the dimensionalities found here are more of a function of the limitations and properties of the models rather than the data, we can make two observations. MDS's optimal dimensionality is capped at 4, even when the data was generated in 10 dimension. This means that MDS needs to simplify the data into much lower dimensions to be able to map them. But, limited number of dimensions could compress or superimpose the variations in the data and reduce the accuracy. On the other hand, Topolow usually adds more dimensions to accommodate the nuances in the data. In the simulated data, all dimensions of the input data hold equal amounts of variance. The added dimensions in the output hold relatively smaller amounts of variance, capturing finer details. Based on the likelihood tests, this property does not affect Topolow's performance, but if the results are to be used to train other models, dimensionality reduction may be beneficial.

Missing	Variant	True Dim	MDS	Topolow
70%	Original	2	2	3
70%	+Noise	2	2	3
70%	+Noise+Bias	2	2	3
85%	Original	2	2	3
85%	+Noise	2	2	4
85%	+Noise+Bias	2	2	3
95%	Original	2	2	4
95%	+Noise	2	2	4
95%	+Noise+Bias	2	2	3
70%	Original	5	4	6
70%	+Noise	5	4	7
70%	+Noise+Bias	5	2	6
85%	Original	5	4	6
85%	+Noise	5	4	20
85%	+Noise+Bias	5	4	10
95%	Original	5	2	15
95%	+Noise	5	2	20
95%	+Noise+Bias	5	2	17
70%	Original	10	4	14
70%	+Noise	10	4	28
70%	+Noise+Bias	10	4	25
85%	Original	10	4	25
85%	+Noise	10	4	29
85%	+Noise+Bias	10	4	20
95%	Original	10	3	10
95%	+Noise	10	3	16
95%	+Noise+Bias	10	3	11

Table S-1. Parameters and characteristics of simulated datasets.

Parameter	Value	Description
Total antigens	250	Split into 150 test and 100 reference antigens
Dimensions	2, 5, 10	Representing different complexity levels
Mean antigenic distances	20.86, 32.60, 46.79	For datasets in dimensions 2, 5, and 10, respectively
SD of antigenic distances	14.16, 19.18, 27.52	For datasets in dimensions 2, 5, and 10, respectively
Missing data proportion	70%, 85%, 95%	Percentage of unmeasured relationships

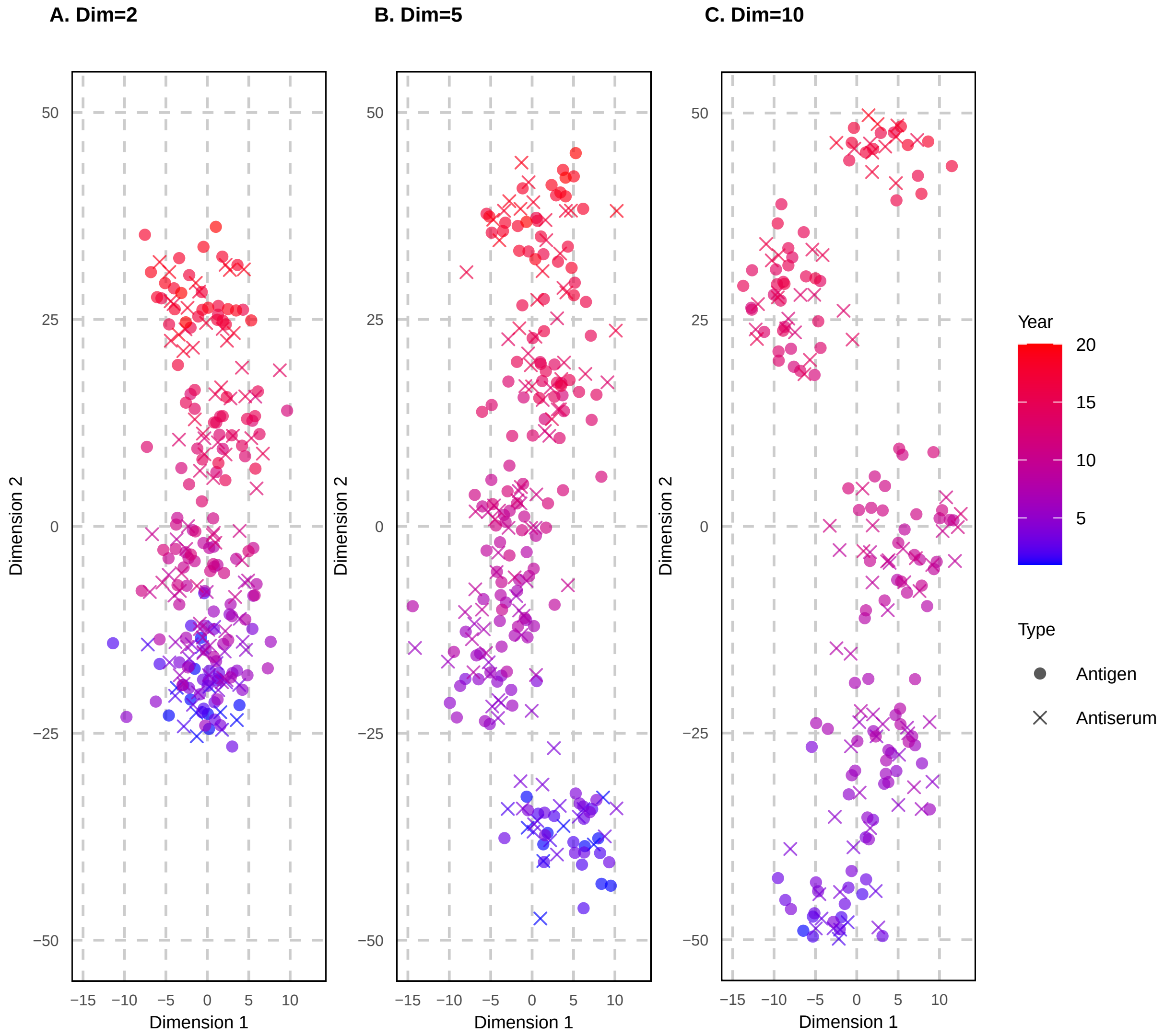

Fig. S-2. 2D projection of three simulated datasets using PCA. Imaginary years 1 to 20 are assigned to points. Note that the differences in trend strength and dispersity observed in 2D projections is a byproduct of the differences in original dimensionalities of datasets.

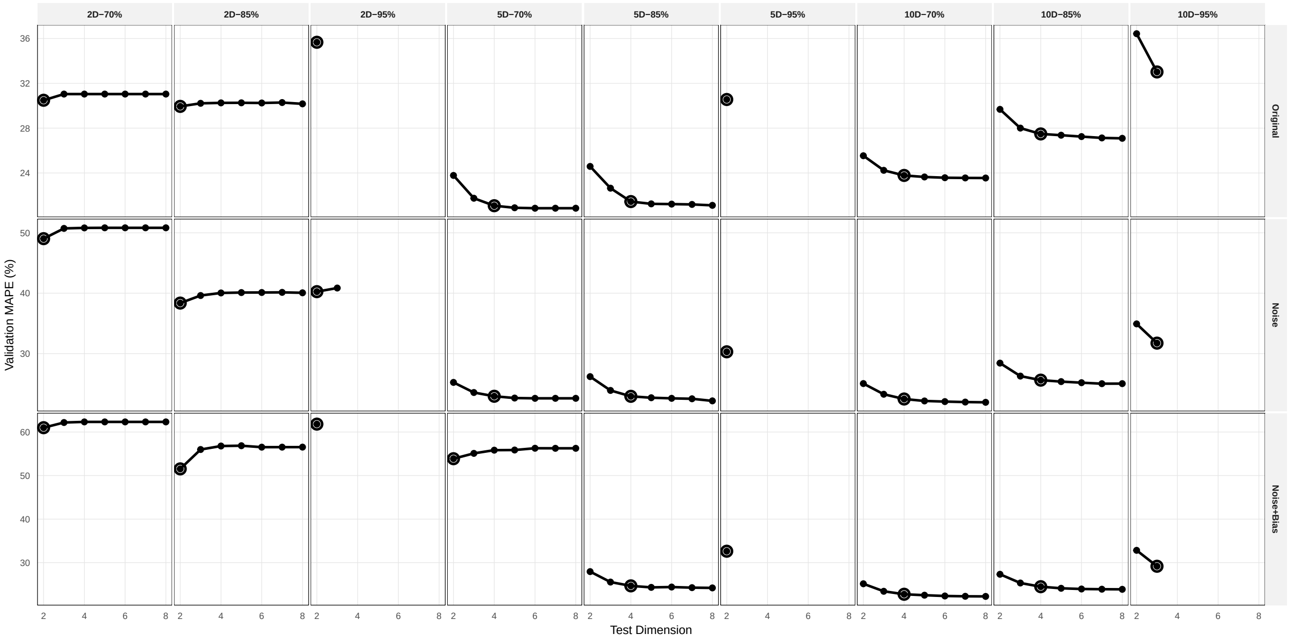

Fig. S-3. Using elbow method to determine the optimal dimensionality for MDS (denoted by circles around dots) working with simulated data generated in 2, 5, and 10 dimensions with different variants of missingness, noise, and bias. The optimal dimensionality was found using the elbow method, conditioned on a completeness > 95% to ensure the usability of maps and keep them comparable with Topolow maps. Completeness was calculated as the number of antigens on the output map divided by the number of antigens in the input data. Although the simulated data were generated in 2, 5, and 10 dimensions, the optimal dimensions determined for MDS never exceeded 4. It is evident that with data having 70% or 85% missingness, MDS holds up the 95% completeness when mapping the data in 2 to 8 dimensions, but fails to do so when missingness is 95% –which is closer to empirical data (91% in H3N2 and 94% in HIV).

Table S-3. Performance across complexity levels and missingness proportions (validation MAPE ± SD). Supporting information for Figure 2

Dim.	Missing	Variant	MDS	Topolow
2	70%	Original	13.436 ± 0.818	0.334 ± 0.164
2	70%	+Noise	12.801 ± 1.028	4.478 ± 0.889
2	70%	+Noise+Bias	12.792 ± 0.955	6.026 ± 0.463
2	85%	Original	18.879 ± 2.419	1.409 ± 1.078
2	85%	+Noise	20.148 ± 3.243	7.976 ± 2.612
2	85%	+Noise+Bias	19.627 ± 1.591	6.938 ± 0.816
2	95%	Original	32.624 ± 5.681	11.252 ± 7.051
2	95%	+Noise	31.872 ± 3.770	20.139 ± 7.988
2	95%	+Noise+Bias	32.806 ± 5.682	16.410 ± 5.163
5	70%	Original	29.657 ± 0.837	0.187 ± 0.050
5	70%	+Noise	27.607 ± 0.711	4.883 ± 0.636
5	70%	+Noise+Bias	29.817 ± 1.372	6.829 ± 0.624
5	85%	Original	34.687 ± 1.518	4.148 ± 2.737
5	85%	+Noise	34.420 ± 1.445	8.559 ± 1.469
5	85%	+Noise+Bias	32.206 ± 1.648	8.068 ± 0.803
5	95%	Original	44.990 ± 3.310	19.065 ± 4.777
5	95%	+Noise	44.544 ± 3.246	21.218 ± 7.206
5	95%	+Noise+Bias	44.422 ± 2.214	17.601 ± 4.297
10	70%	Original	38.584 ± 1.233	1.411 ± 0.525
10	70%	+Noise	35.690 ± 1.343	5.924 ± 0.627
10	70%	+Noise+Bias	35.470 ± 1.410	7.261 ± 0.457
10	85%	Original	45.605 ± 1.366	6.299 ± 0.602
10	85%	+Noise	42.674 ± 1.725	8.353 ± 0.683
10	85%	+Noise+Bias	43.148 ± 2.004	8.498 ± 0.569
10	95%	Original	50.977 ± 3.546	16.176 ± 2.717
10	95%	+Noise	48.604 ± 2.264	18.577 ± 9.932
10	95%	+Noise+Bias	49.014 ± 3.487	15.248 ± 2.644

Table S-4. Paired t-test results between validation MAE of Topolow and MDS over each fold of a 20-fold cross-validation simulation for all scenarios.

Dim.	Missing	Variant	p-value
2	70%	+Noise	<0.0001
2	70%	+Noise+Bias	<0.0001
2	70%	Original	<0.0001
2	85%	+Noise	<0.0001
2	85%	+Noise+Bias	<0.0001
2	85%	Original	<0.0001
2	95%	+Noise	<0.0001
2	95%	+Noise+Bias	<0.0001
2	95%	Original	<0.0001
5	70%	+Noise	<0.0001
5	70%	+Noise+Bias	<0.0001
5	70%	Original	<0.0001
5	85%	+Noise	<0.0001
5	85%	+Noise+Bias	<0.0001
5	85%	Original	<0.0001
5	95%	+Noise	<0.0001
5	95%	+Noise+Bias	<0.0001
5	95%	Original	<0.0001
10	70%	+Noise	<0.0001
10	70%	+Noise+Bias	<0.0001
10	70%	Original	<0.0001
10	85%	+Noise	<0.0001
10	85%	+Noise+Bias	<0.0001
10	85%	Original	<0.0001
10	95%	+Noise	<0.0001
10	95%	+Noise+Bias	<0.0001
10	95%	Original	<0.0001

Table S-5. Paired t-tests between validation MAE of Topolow and MDS over a 20-fold cross-validation on empirical data indicate that errors in Topolow are significantly smaller than in MDS. This is a consistent pattern across datasets with diverse characteristics, from HIV –with 335 antigens and the highest missing proportion– to DENV –with only 47 antigens and the most complete data. Each algorithm was run with its optimal dimensions for the data. The missing proportion of the input data (in the matrix of all titers) is shown in the table.

Dataset	Missing	p-value
Influenza (H3N2)	91%	<0.0001
HIV (B & C)	94%	<0.0001
DENV (serotypes 1, 2, 3, 4)	77%	<0.0001

Table S-6. Count of mapped antigens with simulated data. Nine datasets were created in dimensions 2, 5, and 10 (column Dim) and 3 levels of proportions of data were removed: 70%, 85%, and 95% (column Missing) –the same as the scenarios explained in the main text. For each scenario, the optimal dimensionality for mapping was found through likelihood maximization using Topolow, then the data was mapped by MDS (column MDS Opt. Dim.) and Topolow (column Topolow) in the same optimal dimensions. MDS was also ran with 2 dimensions because it is the dimensionality where MDS consistently places >95% of points in maps –an important usefulness criterion. However, errors in 2D may be sub-optimal.

Dim	Missing	MDS 2D	MDS Opt. Dim.	Topolow
2	70%	250	250	250
2	85%	250	250	250
2	95%	**246**	**240**	250
5	70%	250	250	250
5	85%	250	250	250
5	95%	**248**	**30**	250
10	70%	250	**188**	250
10	85%	250	**112**	250
10	95%	**245**	**3**	250

Table S-7. Distribution of validation errors (Mean ± SD) over the simulated scenarios. Mean values quantify the bias in estimates by each method.

Dim.	Missing	Variant	MDS	Topolow
2	70%	+Noise	1.105 ± 1.081	-0.043 ± 0.334
2	70%	+Noise+Bias	1.204 ± 0.973	0.528 ± 0.425
2	70%	Original	1.313 ± 1.056	-0.006 ± 0.040
2	85%	+Noise	1.379 ± 1.421	-0.093 ± 0.479
2	85%	+Noise+Bias	1.470 ± 1.389	0.426 ± 0.482
2	85%	Original	1.513 ± 1.333	-0.021 ± 0.256
2	95%	+Noise	1.805 ± 2.197	-0.303 ± 1.383
2	95%	+Noise+Bias	1.720 ± 2.238	0.135 ± 1.353
2	95%	Original	1.937 ± 2.208	-0.230 ± 1.118
5	70%	+Noise	4.869 ± 2.522	-0.085 ± 0.919
5	70%	+Noise+Bias	5.067 ± 2.702	0.997 ± 0.930
5	70%	Original	5.245 ± 2.410	-0.004 ± 0.065
5	85%	+Noise	5.538 ± 2.856	-0.289 ± 1.313
5	85%	+Noise+Bias	5.237 ± 2.735	0.682 ± 1.288
5	85%	Original	5.640 ± 2.756	-0.049 ± 0.958
5	95%	+Noise	6.120 ± 3.848	-1.046 ± 5.730
5	95%	+Noise+Bias	6.043 ± 3.952	-0.074 ± 3.246
5	95%	Original	6.129 ± 3.742	-0.782 ± 4.249
10	70%	+Noise	8.966 ± 2.863	-0.172 ± 1.574
10	70%	+Noise+Bias	8.954 ± 2.918	1.368 ± 1.596
10	70%	Original	9.733 ± 2.887	-0.024 ± 0.490
10	85%	+Noise	9.717 ± 3.232	-0.345 ± 2.033
10	85%	+Noise+Bias	9.814 ± 3.306	1.100 ± 1.970
10	85%	Original	10.326 ± 3.201	-0.109 ± 1.609
10	95%	+Noise	9.685 ± 4.485	-1.146 ± 6.935
10	95%	+Noise+Bias	9.750 ± 4.459	0.366 ± 3.888
10	95%	Original	10.191 ± 4.217	-0.439 ± 3.986

Table S-8. Comparison of MAPE (%) in the inputs due to added noise/bias against MAPE of locations determined by MDS and Topolow. All errors are calculated against the known non-noisy ground truth.

Dim.	Missing	Variant	Input	MDS	Topolow
2	70%	+Noise	8.046	12.630	3.764
2	70%	+Noise+Bias	11.121	12.723	5.895
2	85%	+Noise	7.888	19.133	4.496
2	85%	+Noise+Bias	11.133	18.821	6.243
2	95%	+Noise	7.141	30.696	5.872
2	95%	+Noise+Bias	10.183	29.355	7.162
5	70%	+Noise	5.693	29.917	3.205
5	70%	+Noise+Bias	8.488	29.769	6.507
5	85%	+Noise	5.420	37.041	4.073
5	85%	+Noise+Bias	7.867	35.523	6.328
5	95%	+Noise	4.791	48.159	5.459
5	95%	+Noise+Bias	7.173	47.703	7.324
10	70%	+Noise	5.411	37.265	3.825
10	70%	+Noise+Bias	7.989	37.121	6.504
10	85%	+Noise	5.170	44.970	4.559
10	85%	+Noise+Bias	7.507	45.315	6.634
10	95%	+Noise	4.855	53.920	5.343
10	95%	+Noise+Bias	6.900	53.654	6.910

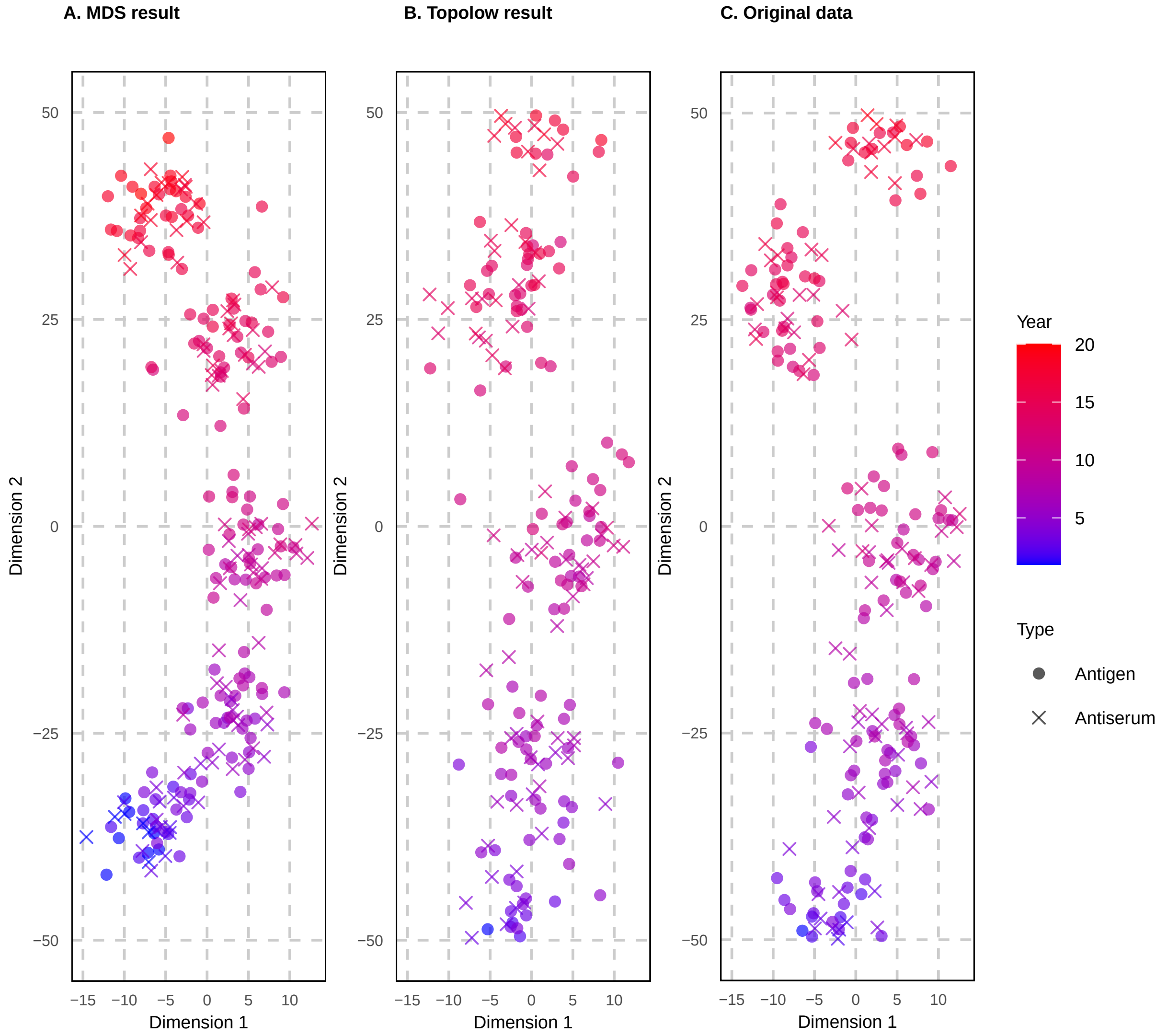

Fig. S-4. Antigenic maps for simulated scenario with 10 dimensions, 95% missing distances, and added noise and bias using (**A**) MDS and (**B**) Topolow compared to the 2D projection of the original data (**C**).

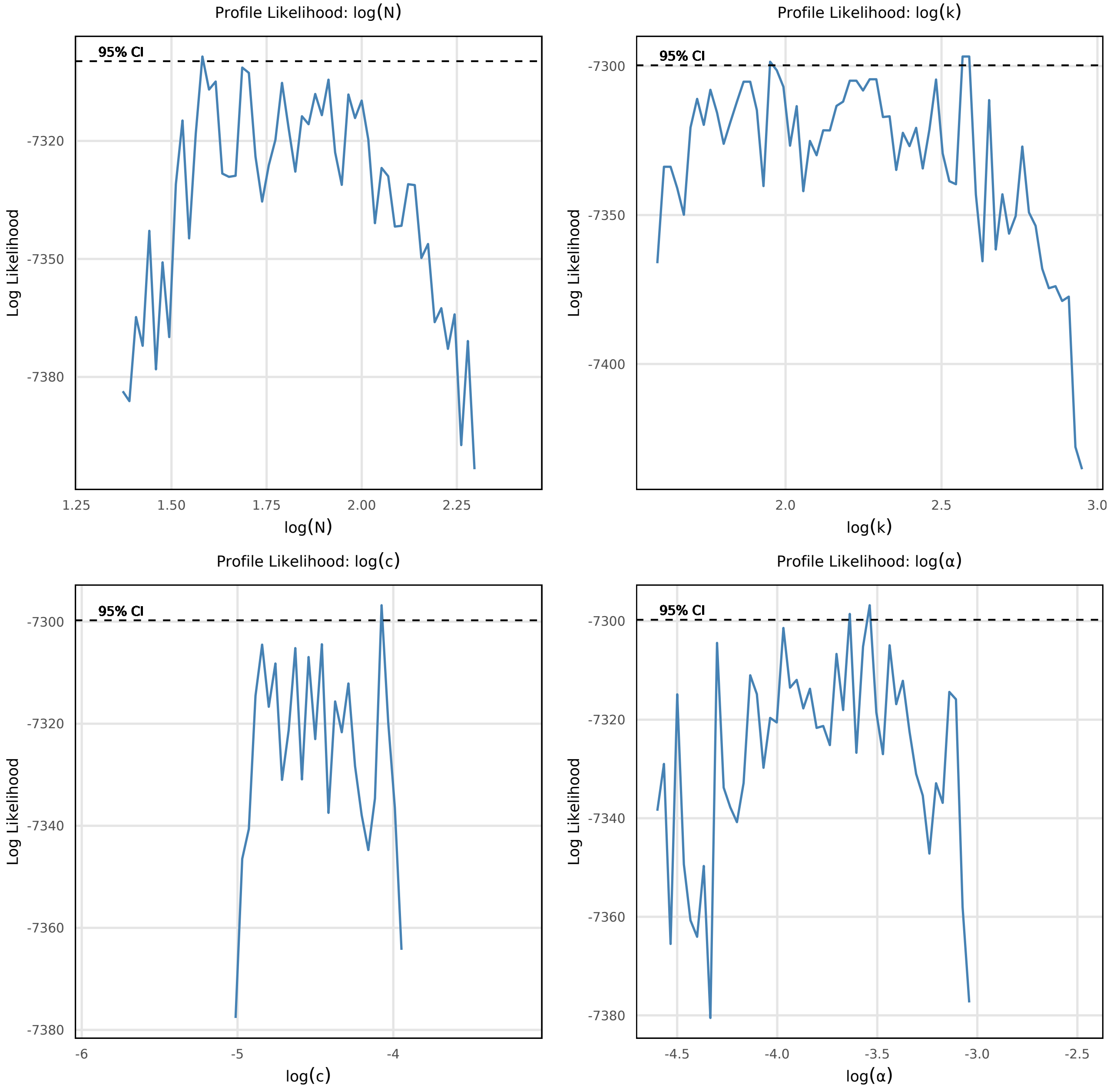

Fig. S-5. Empirical profile likelihood plots for model parameters with H3N2 dataset. Profile likelihood for (a) dimensionality parameter N, (b) initial spring constant k, (c) repulsion constant c, and (d) cooling rate α, showing optimal values and 95% confidence intervals calculated from likelihood ratio test.

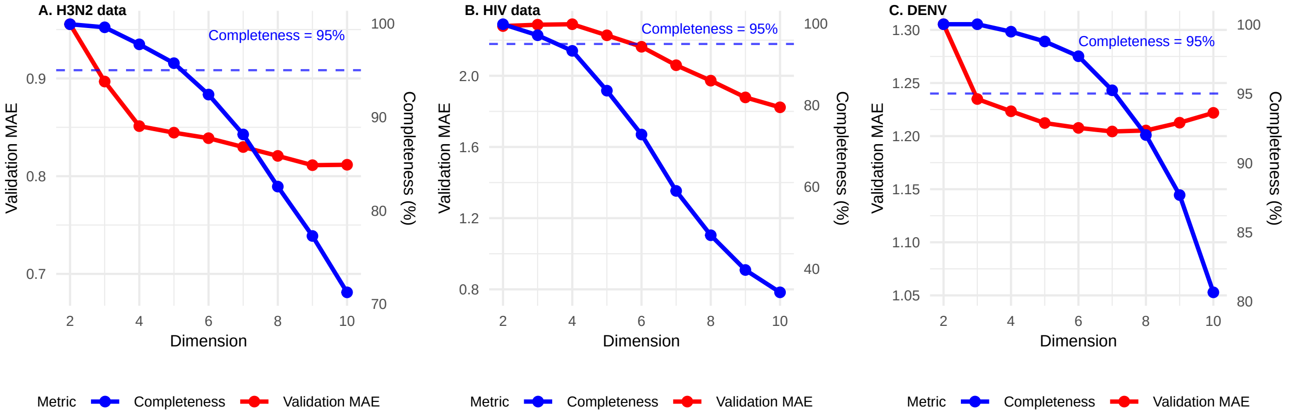

Fig. S-6. Validation MAE and completeness (coverage) of MDS maps in different dimensions. (**A**) shows H3N2, (**B**) HIV, and (**C**) DENV data. Completeness was calculated as the number of antigens on the output map divided by the number of antigens in the input data. The optimal dimensionality was determined through finding the *elbow* of MAE curve, conditioned on a completeness $> 95\%$ to ensure the usability of maps and keep them comparable with Topolow maps. 4 dimensions for H3N2, 2 dimensions for HIV, and 3 dimensions for DENV data were chosen.

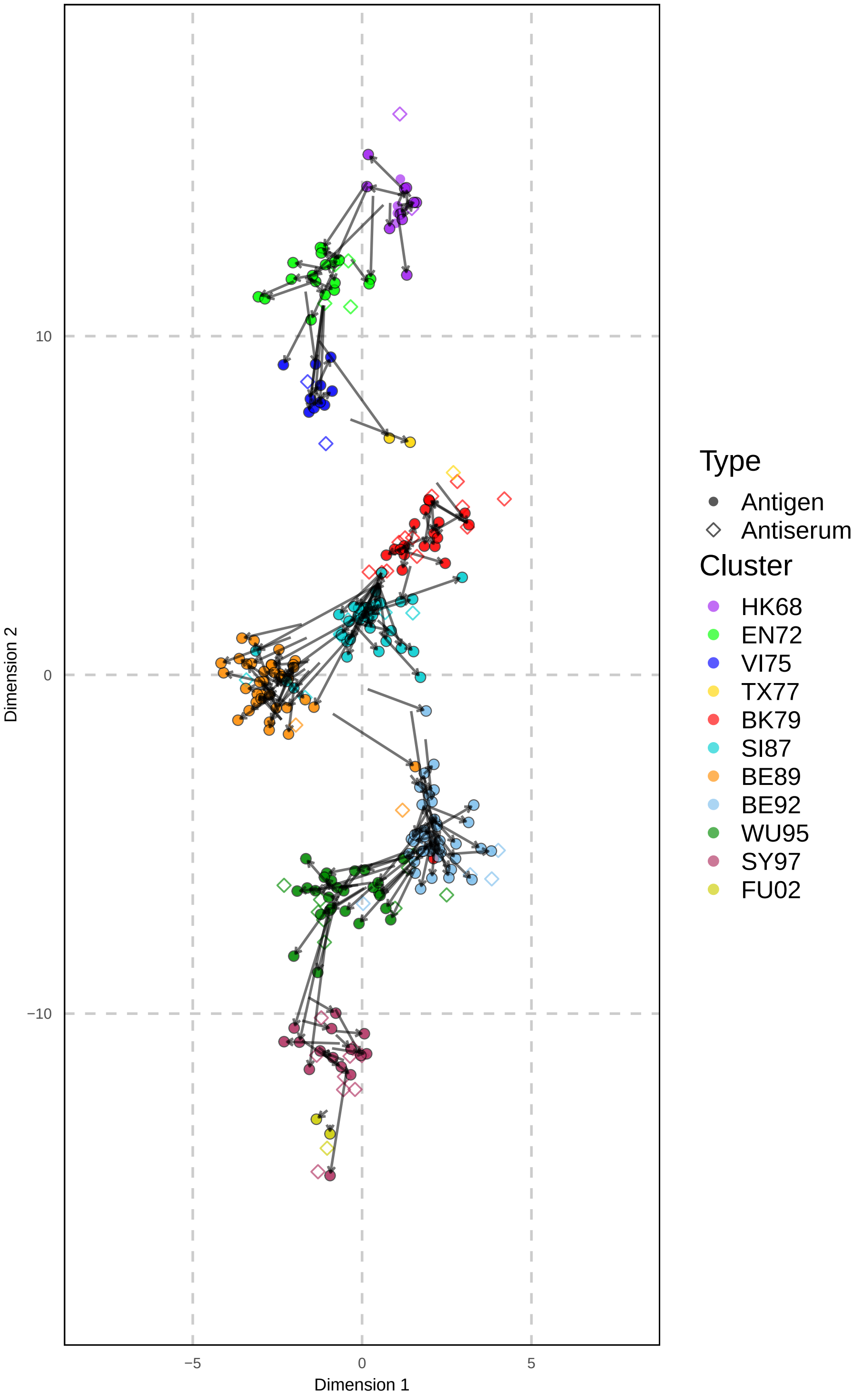

Fig. S-7. Antigenic map estimated by Topolow for H3N2 HI titers. Test antigens are shown as colored circles and reference antigens as colored diamonds, with colors denoting antigens' clusters inferred by Smith et al. (2004). All antigenic velocity vectors are shown on the map. $\sigma_t = 1.13$ years, $\sigma_{ag} = 1.8$-fold, and clade depth of 5 edges on the phylogenetic tree were the optimal values found for antigenic velocity calculations. Each unit on the map corresponds to a 2-fold change in HI titer.

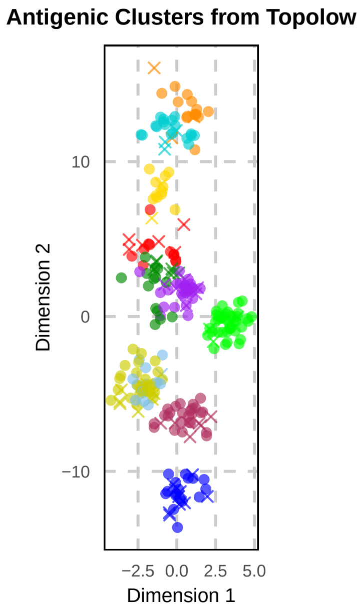

Fig. S-8. Clustering of the coordinates found by Topolow in the optimal dimensionality (4D) for H3N2 data using K-means algorithm. The points are projected into 2D space using PCA. Colors separate clusters and have no specific label. It is noteworthy that the groupings in the original dimensionality do not match with the groupings one would expect based on the proximities in the 2D space.

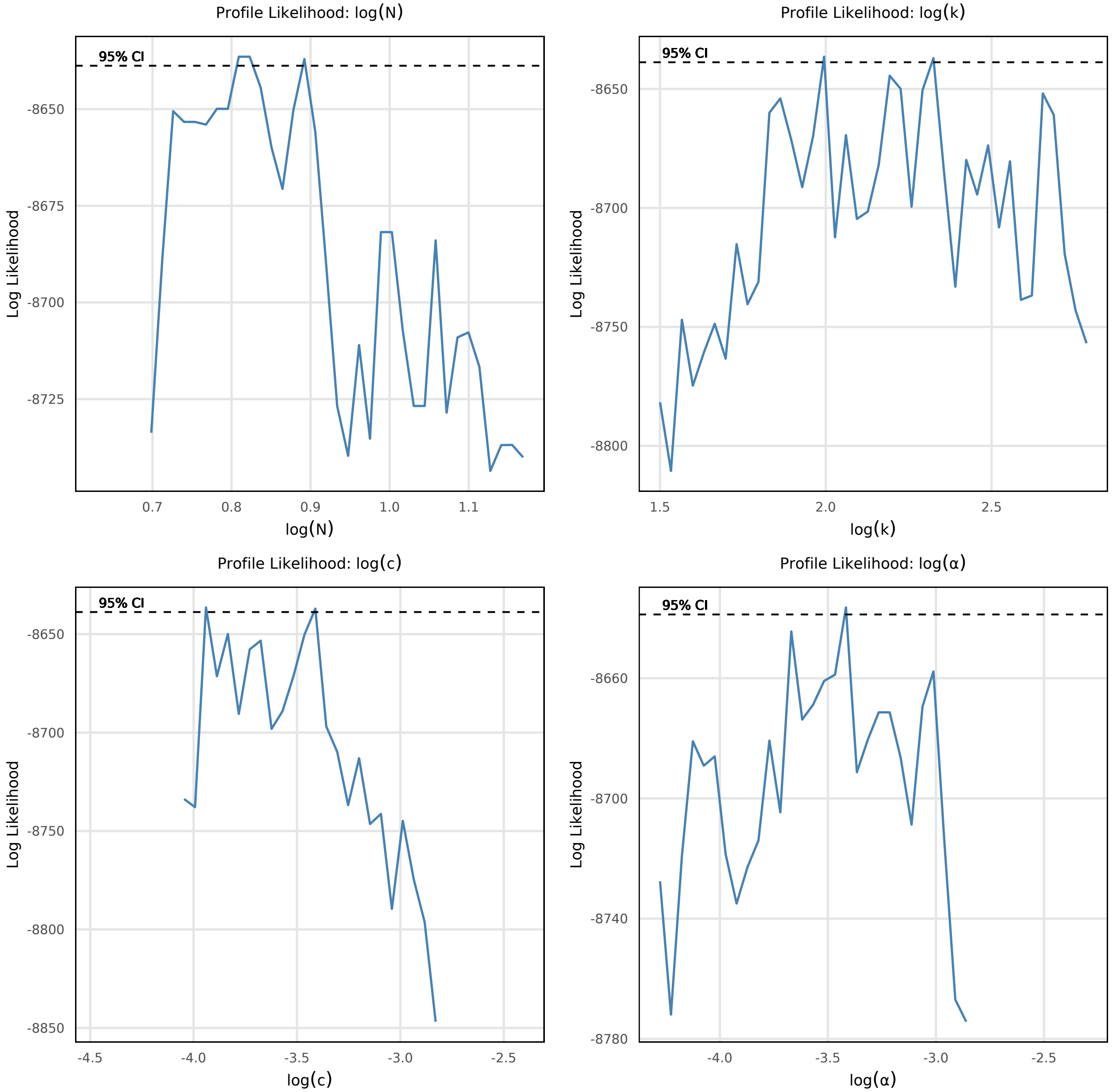

Fig. S-9. Empirical profile likelihood plots for model parameters with HIV dataset. Profile likelihood for (a) dimensionality parameter N, (b) initial spring constant k, (c) repulsion constant c, and (d) cooling rate α, showing optimal values and 95% confidence intervals calculated from likelihood ratio test.

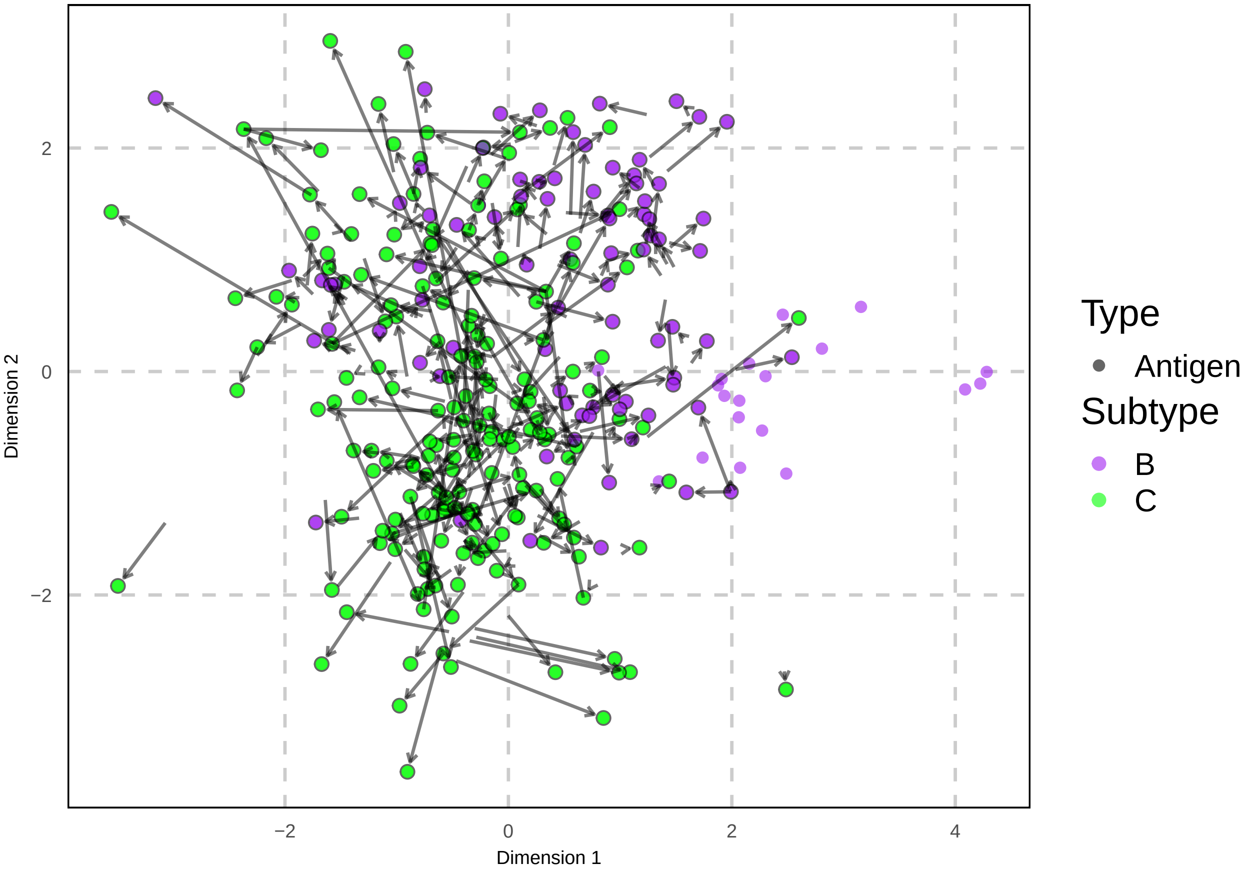

Fig. S-10. Antigenic map estimated by Topolow for HIV-1 subtype B and C viruses. Only test antigens are shown as colored circles (reference antigens are not shown to keep the figure more readable). All antigenic velocity vectors are shown on the map, except for antigens with empty neighborhoods. $\sigma_t = 0.98$ year, $\sigma_{ag} = 0.43$-fold, and clade depth of 4 edges on the phylogenetic tree were the optimal values found for antigenic velocity calculations. Each unit on the map corresponds to a 2-fold change in neutralization titer.

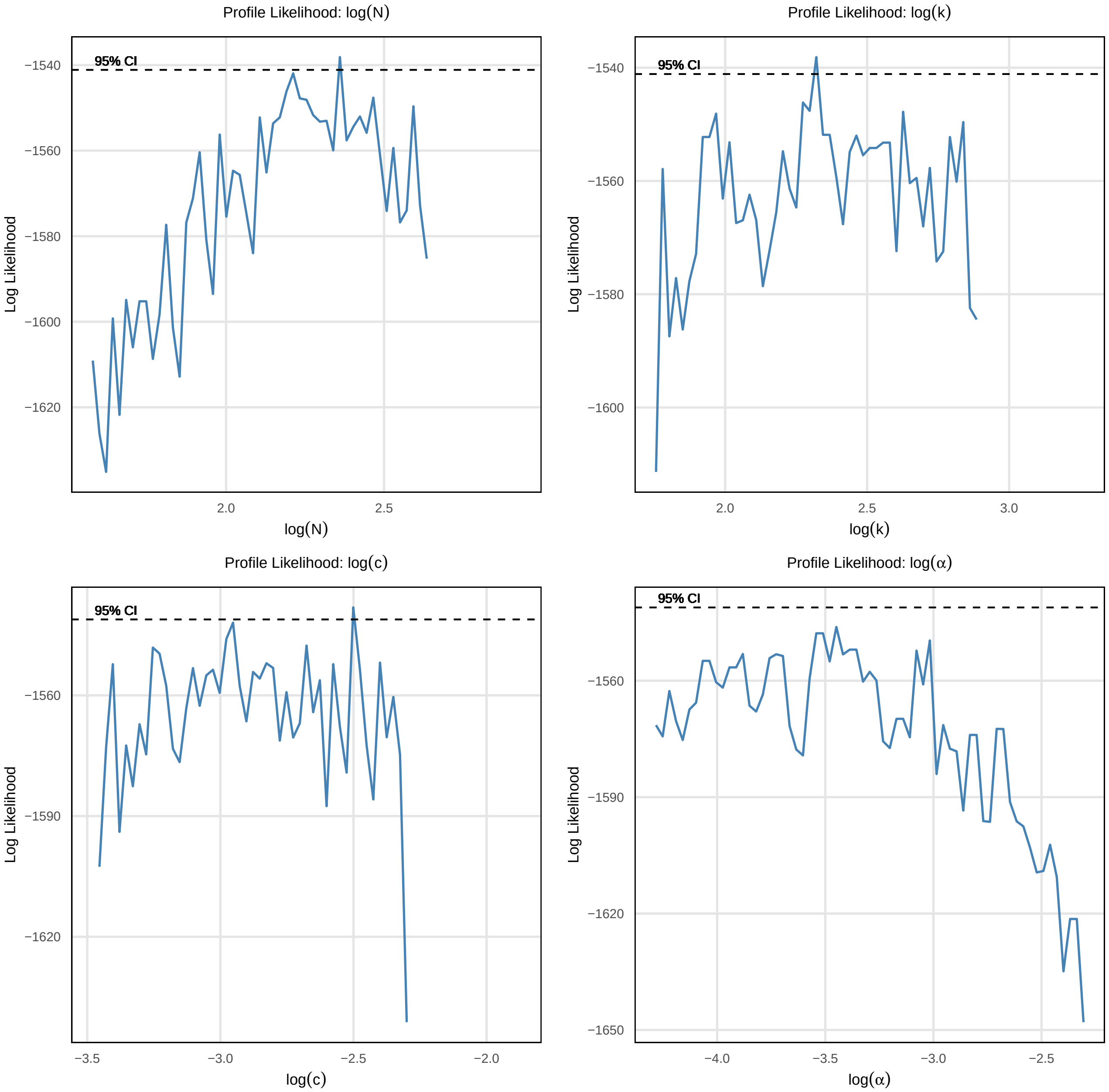

Fig. S-11. Empirical profile likelihood plots for model parameters with DENV dataset. Profile likelihood for (a) dimensionality parameter N, (b) initial spring constant k, (c) repulsion constant c, and (d) cooling rate α, showing optimal values and 95% confidence intervals calculated from likelihood ratio test.

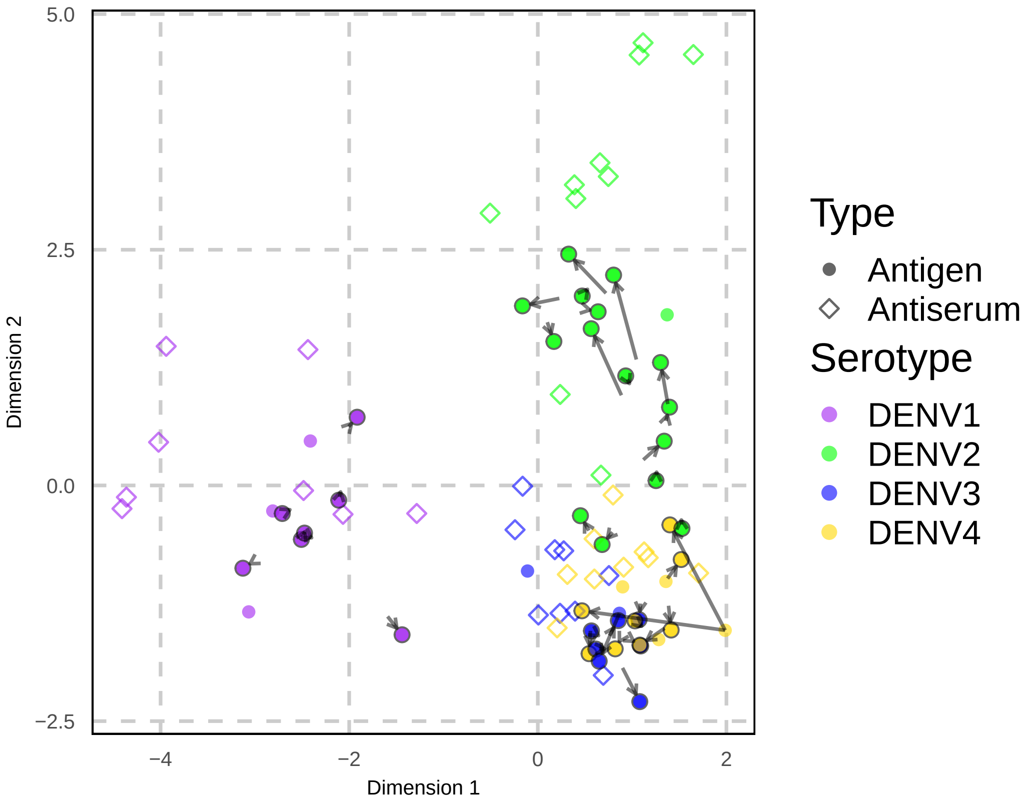

Fig. S-12. Antigenic map estimated by Topolow for DENV viruses. Test antigens are shown as colored circles and reference antigens as colored diamonds, with colors denoting serotypes. All antigenic velocity vectors are shown on the map, except for antigens with empty neighborhoods. $\sigma_t = 4.12$ year, $\sigma_{ag} = 0.55$-fold, and clade depth of 3 edges on the phylogenetic tree were the optimal values found for antigenic velocity calculations. Each unit on the map corresponds to a 2-fold change in neutralization titer.

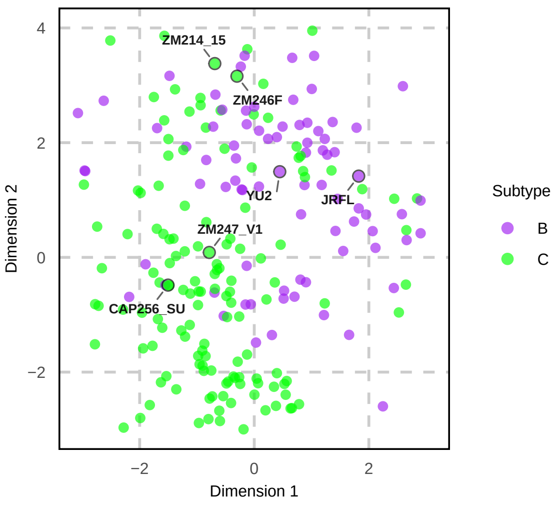

Fig. S-13. 2D visualization of HIV antigenic map created by MDS (RACMACS), colored by subtypes. It is notable that the well-characterized JRCSF antigen is absent from the MDS map, and two important subtype C references, ZM214 15 and ZM246F, appear distant from the majority of subtype C isolates. Although the cluster formation is not visually too different from the map produced by Topolow, error measurements in Fig. 3 show 41% decrease in validation MAE by Topolow.

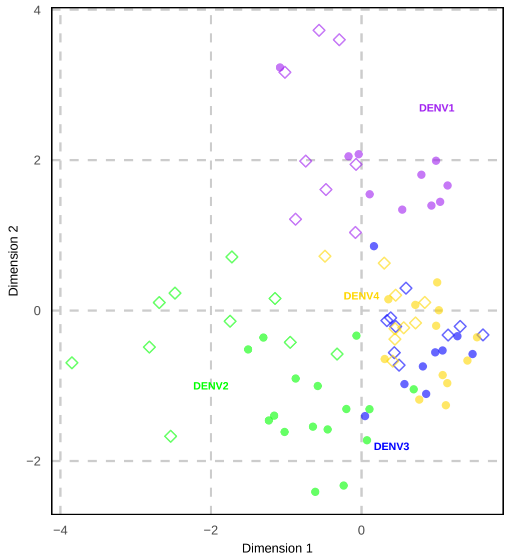

Fig. S-14. 2D visualization of DENV antigenic map created by MDS (RACMACS), colored by serotypes. Although the cluster formation is not visually too different from the map produced by Topolow, error measurements in Fig. 3 show 56% decrease in validation MAE by Topolow.

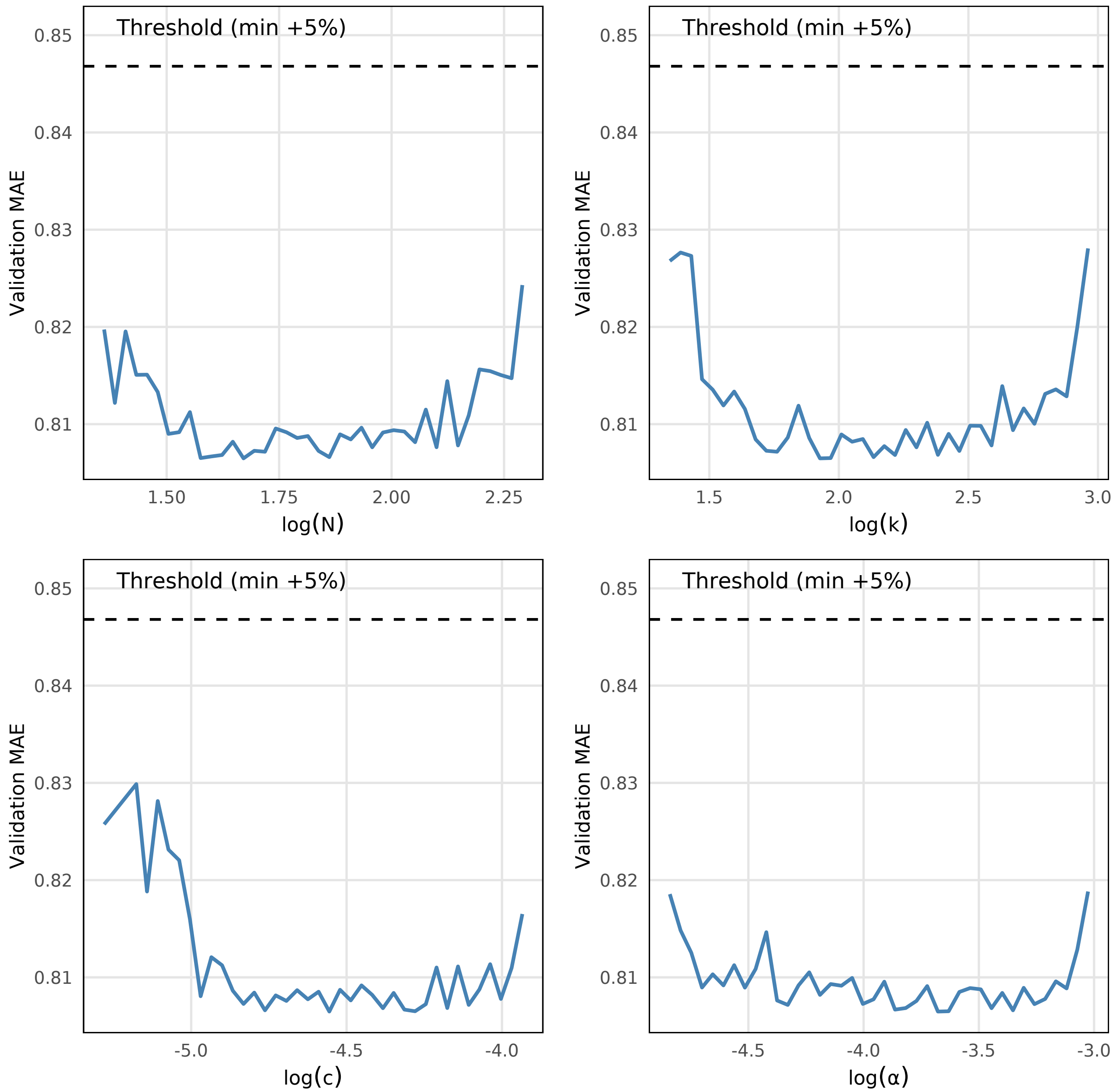

Fig. S-15. Empirical parameter sensitivity plots for model parameters in terms of validation MAE on H3N2 dataset. To find the validation MAE at each value of a parameter, the optimal values of other parameters which lead to the minimum validation MAE at that point were used. (a) dimensionality parameter N, (b) initial spring constant k, (c) repulsion constant c, and (d) cooling rate α. 5% above the minimum MAE is shown as an acceptable threshold. Error is in the acceptable range for a wide range of parameter values. For comparison, validation MAE of MDS for this data is 0.836.

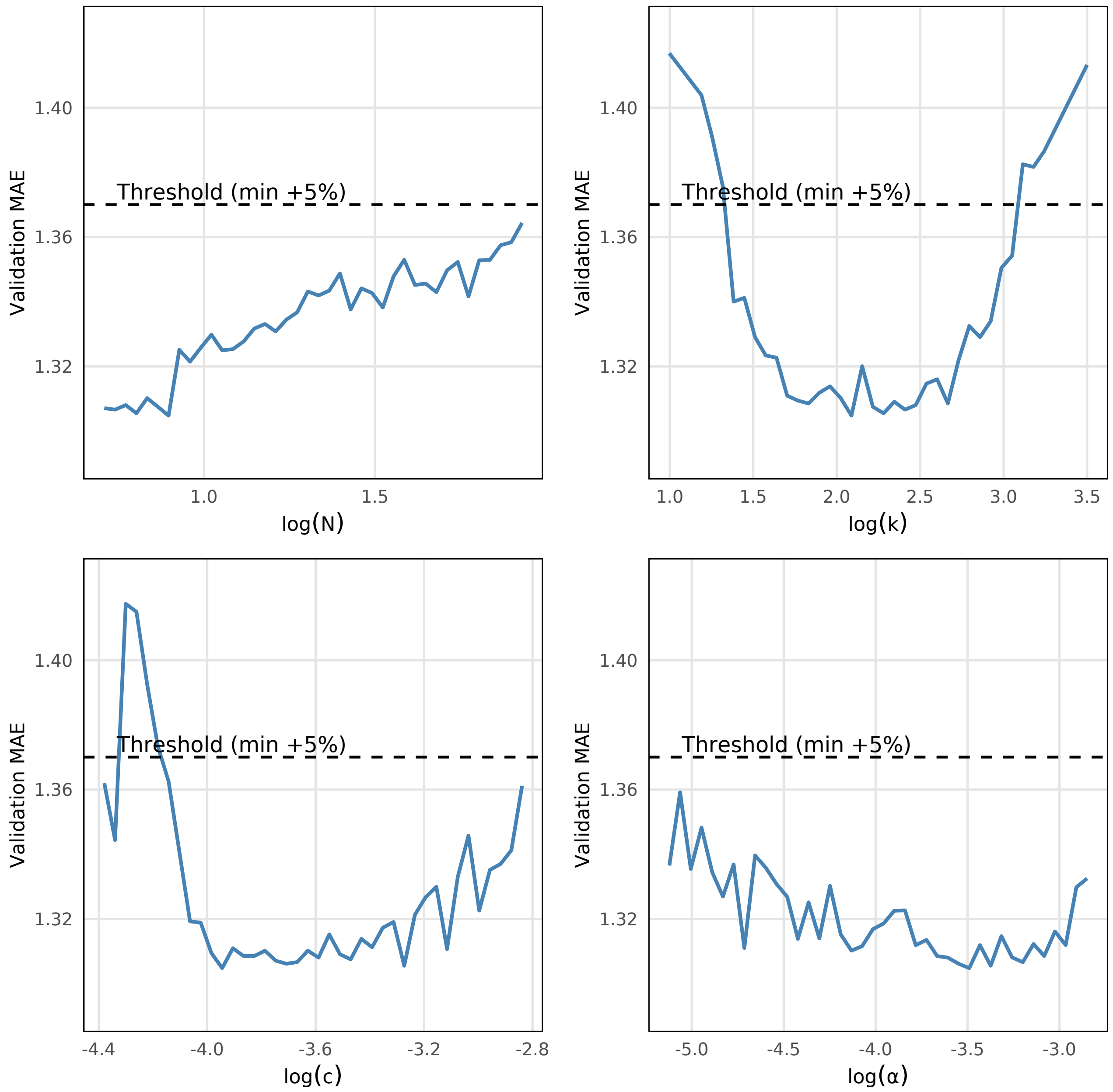

Fig. S-16. Empirical parameter sensitivity plots for model parameters in terms of validation MAE on HIV dataset. To find the validation MAE at each value of a parameter, the optimal values of other parameters which lead to the minimum validation MAE at that point were used. (a) dimensionality parameter N, (b) initial spring constant k, (c) repulsion constant c, and (d) cooling rate α. 5% above the minimum MAE is shown as an acceptable threshold. Error is in the acceptable range for a wide range of parameter values. For comparison, validation MAE of MDS for this data is 2.214.

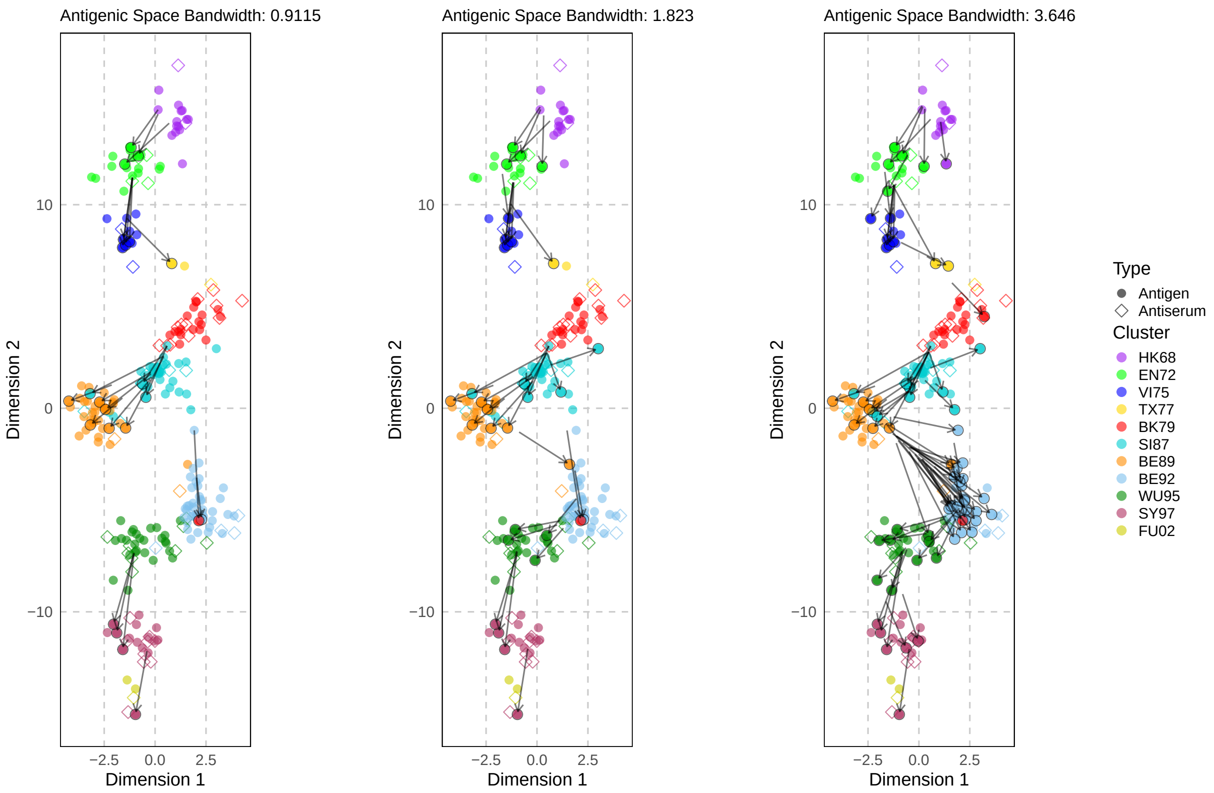

Fig. S-17. Maps with antigenic velocity vectors (longer than 2-fold) for H3N2 dataset with various antigenic space bandwidths (σ_{ag}). σ_{ag}=1.823 in the middle is the optimal data-driven value found by Silverman's rule.
A Large σ_{ag} (assuming σ_t and clade depth being constant) expands the background to more antigenically different samples, leading to longer velocity vectors for more samples.

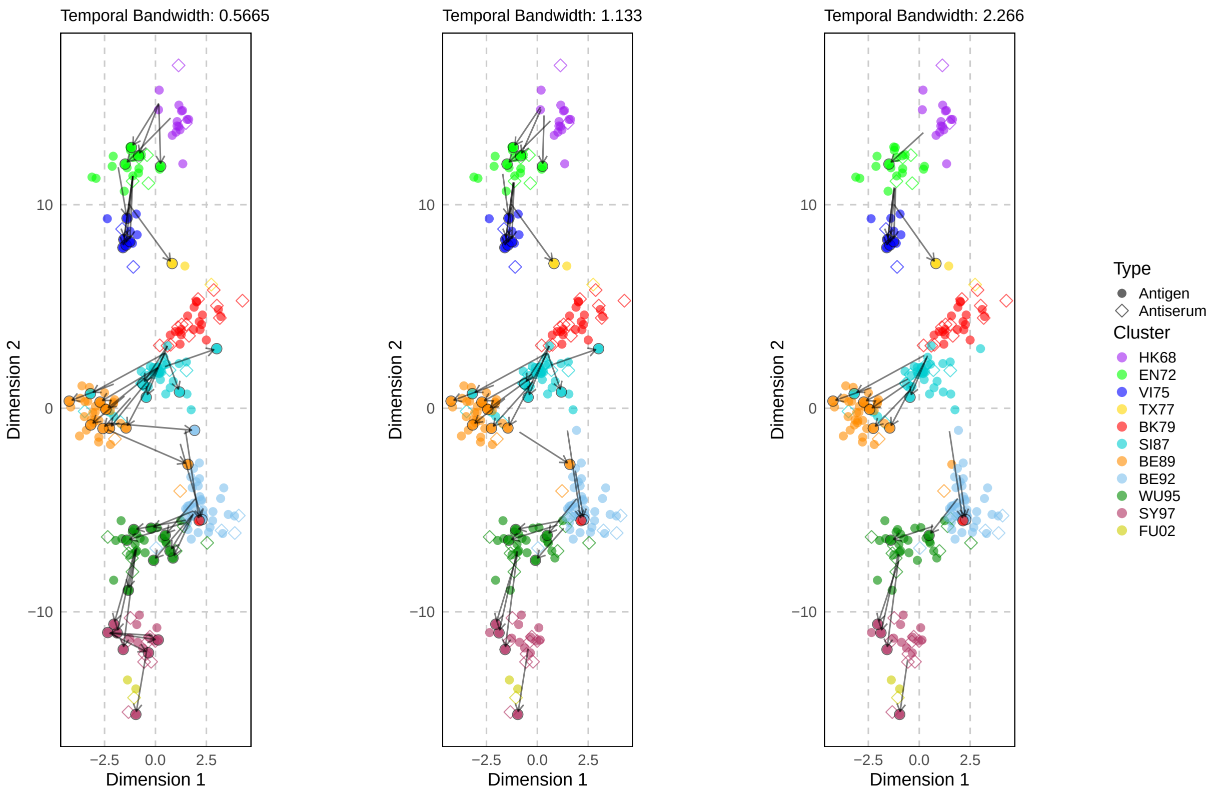

Fig. S-18. Maps with antigenic velocity vectors (longer than 2-fold) for H3N2 dataset with various temporal bandwidths (σ_t). σ_t=1.133 in the middle is the optimal data-driven value found by Silverman's rule.
A large σ_t (assuming σ_{ag} and clade depth being constant) expands the background to older samples in the same neighborhood in the antigenic space. Therefore, in $\frac{\mathbf{x}_i - \mathbf{x}_j}{t_i - t_j}$ terms in Eq. 12 denominator potentially increases while numerator is limited by σ_{ag}. In other words, a larger σ_t has the potential to add older samples to the equation, diluting the velocity.

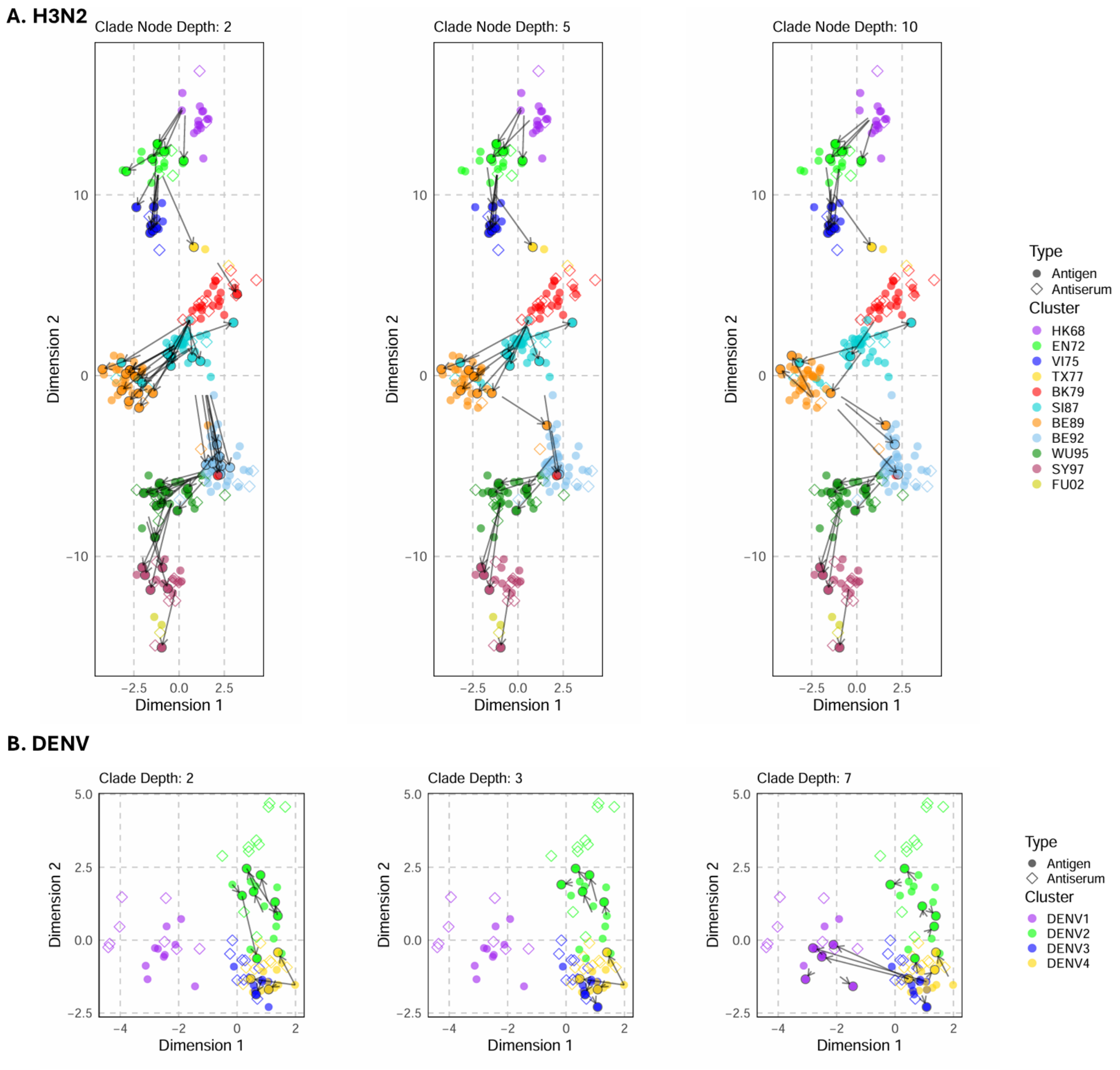

Fig. S-19. Maps with antigenic velocity vectors with various clade depth cutoffs. (**A**) Shows arrows longer than 2-fold for H3N2 data. (**B**) Shows arrows longer than 0.3-fold for DENV data. The middle column is the optimal data-driven clade depth found by ALBD.

A small clade depth focuses the antigenic velocity calculations on more similar samples, leading to vectors showing within-cluster or local trends. While a large clade depth focuses on long-distance or between-cluster changes. The depth automatically determined from the tree shape (middle column) gives the best balance of local and global evolution. Compare the effect on H3N2 –a virus with semi-linear antigenic advance over time– and DENV –a virus with 4 different concurrent serotypes.

References

Colijn, C. and Gardy, J. (2014). Phylogenetic tree shapes resolve disease transmission patterns. *Evolution, Medicine, and Public Health*, 2014(1):96–108.

Colless, D. (1982). Review of phylogenetics: The theory and practice of phylogenetic systematics. *Systematic Zoology*, 31(1):100–104.

Goddard, W. and Oellermann, O. (2011). Distance in graphs. In Dehmer, M., editor, *Structural Analysis of Complex Networks*, pages 49–72. Birkhäuser Boston.

Gundersen, M. and Vadstein, O. (2024). An exploration of the impact of phylogenetic tree structure on nti and βnti estimates of community assembly. *Scientific Reports*, 14:23480.

Petrie, J., Ohmit, S., Johnson, E., Truscon, R., and Monto, A. (2015). Persistence of antibodies to influenza hemagglutinin and neuraminidase following one or two years of influenza vaccination. *The Journal of Infectious Diseases*, 212(12):1914–1922.

Sackin, M. (1972). Evolutionary trees and the unevenness of evolution. *Taxon*, 21:117–119.

Silverman, B. (1986). *Density Estimation for Statistics and Data Analysis*. Chapman & Hall.

Smith, D., Lapedes, A., de Jong, J., Bestebroer, T., Rimmelzwaan, G., Osterhaus, A., and Fouchier, R. (2004). Mapping the antigenic and genetic evolution of influenza virus. *Science*, 305(5682):371–376.

Wand, M. and Jones, M. (1995). *Kernel Smoothing*. Chapman & Hall.

Wilks, S. (2022). Racmacs.
